# Magallanes: a web services discovery and automatic workflow composition tool

**DOI:** 10.1186/1471-2105-10-334

**Published:** 2009-10-15

**Authors:** Javier Ríos, Johan Karlsson, Oswaldo Trelles

**Affiliations:** 1 Computer Architecture Department, University of Malaga, 29080, Malaga, Spain

## Abstract

**Background:**

To aid in bioinformatics data processing and analysis, an increasing number of web-based applications are being deployed. Although this is a positive circumstance in general, the proliferation of tools makes it difficult to find the right tool, or more importantly, the right set of tools that can work together to solve real complex problems.

**Results:**

Magallanes (Magellan) is a versatile, platform-independent Java library of algorithms aimed at discovering bioinformatics web services and associated data types. A second important feature of Magallanes is its ability to connect available and compatible web services into workflows that can process data sequentially to reach a desired output given a particular input. Magallanes' capabilities can be exploited both as an API or directly accessed through a graphic user interface.

The Magallanes' API is freely available for academic use, and together with Magallanes application has been tested in MS-Windows™ XP and Unix-like operating systems. Detailed implementation information, including user manuals and tutorials, is available at .

**Conclusion:**

Different implementations of the same client (web page, desktop applications, web services, etc.) have been deployed and are currently in use in real installations such as the National Institute of Bioinformatics (Spain) and the ACGT-EU project. This shows the potential utility and versatility of the software library, including the integration of novel tools in the domain and with strong evidences in the line of facilitate the automatic discovering and composition of workflows.

## Background

Applications and databases available online for bioinformatics research are rapidly proliferating [1]; however, the absence of effective discovery tools for these resources prevents them from being combined in workflows to create powerful bioinformatics machines.

Typically, in service-oriented architectures, dynamic discovery of tools is made possible by registering tool metadata in a shared repository or registry--for example, UDDI. In bioinformatics, some metadata repositories such as BioMoby [2] and FETA [3] also recognize the importance of sharing data formats between tools, and make use of this strategy to implement integration architectures. Such repository approaches have collected large sets of registered services and data types, making a manual discovery process difficult and time consuming. Support for this task has become crucial.

In general, a discovery process aims to segregate a set of services or data-types that satisfy a given number of requirements from the larger pool of available resources; for example, what services are able to process my molecular sequence?

These discovery processes can be based on syntax or semantics. In bioinformatics, a syntax-based discovery process is often unsatisfactory because it presumes knowledge of the names of the objects or services to be searched. Semantics-based discovery processes enable a more accurate discovery mechanism since the descriptions are generally structured and well defined. Magallanes uses a syntactic approach for text-based searches and a semantic approach to combine different services, guided by semantic knowledge about the input and output data types.

The essential task is to design and implement a search engine adapted to the demands of bioinformatics. In this document we present a programmatic library called Magallanes that provides necessary support for flexible and expandable discovery of services, which in turn simplifies the creation of workflows. Results from Magallanes' discovery process can be used as input for workflow generation because web services are automatically combined based on semantic descriptions (specifically based on their input and output data types).

As a proof-of concept, we have developed several variations of the same client (both standalone and web applications) that use Magallanes as a discovery engine (see Results section). These clients can also be embedded in third-party applications.

## Implementation

### Related work

In this section, we outline related work. Since our work consists of a software library which can be re-used in other client software, we look at two aspects: search and workflow composition functionality in existing clients. Although Magallanes support standard WSDL web services, our efforts so far have focused on BioMoby services since there is a large set of services to search and those services are easily composed due to a shared data type ontology.

Therefore, we selected the most prominent clients for BioMoby web services for this overview of related work.

### Service discovery by clients

BioMoby-compatible clients, such as MOWServ [4], Seahawk [5], Remora [6] and Taverna [7], provide support for service searches in various degrees (see complementary material). Typically, services are located by specifying an input data type (which returns compatible services) or by name (partial matches).

BioCatalogue [8] is a public curated catalogue of life science web services that provides a keyword based search and allows filtering the results by some metadata like service type, provider, country, etc. BioCatalogue could benefit from the functionality of Magallanes.

### Automatic workflow composition

GBrowse [9] and Seahawk [5] are two end-user oriented clients with a data-centric approach. Users either specify input data by submitting a data file which the application analyses to determine the correct data type (Seahawk) or they specify identifiers of a sequence in an external database (Seahawk/GBrowse). Available services to further process data are presented once the user data and data type is known to the applications. Seahawk has a slightly more sophisticated approach that lets the user specify what he/she wants to do with the data (using semantic keywords used for by the compatible services) instead of choosing the next service specifically. Both applications allow users to save their results as Taverna workflows.

The strategy in [10] is to simplify interactive service composition of BioMoby services. In each step of the workflow construction process, only those services that are compatible and more likely to be useful are displayed. This is achieved by ranking the services according to several aspects, such as semantic similarity of data type inputs; or by non-functional measurements such as number of retrievals of service definitions from MobyCentral. Their composition algorithm aims to limit the number of services presented to the user. The algorithm considers not only direct compatibility and polymorphism (data type compatibility by inheritance), but also includes services whose input/output data type matches the requested data type either directly or recursively. Results are further ranked by their popularity as measured by requests to the central registry, and by the disparity between data types in the ontology.

### Summarizing the related work

As is evident from section 'Service discovery by clients', search functionality in clients is heterogeneous. Users would benefit from having a standardized way of locating services. Client software developers could, instead of re-inventing search algorithms, focus on providing a user-friendly graphical interface. This shows the need for a freely shared software library.

Regarding workflow generation, authors in [10] do not address the problem of locating data types as input to the workflow generation algorithm. This problem becomes serious considering the proliferation of data types in BioMoby (currently over 800).

While there is clearly a need for service composition support, current approaches fail to recognize the difficulties in a) find initial data type and b) automatically creating an initial version of a workflow. Therefore, we have addressed both a) and b).

### Magallanes' architecture

Existing workflow composition approaches do not assist the user during initial selection of input and output data types, even though this step can be quite complicated. Magallanes aims to do two things: simplify the discovery task, and integrate discovery with composition.

Magallanes consists of a Java library with algorithms and data handling routines built using the Modular API [11]. The Modular API uses specific wrappers called *access*es to map different data types and web-services repositories into a unified model (e.g., parsing the WSDL to get the web service's description, name, etc.). Magallanes can access and manage various remote repositories using a standardized interface, and benefits from a cache system to reduce processing time. Currently, the Modular API can access to BioMoby, INB, ACGT and standard WDSL repositories. In order to support another repository, a new access must be implemented (e.g. we are currently working to incorporate BioCatalogue in the list of available repositories).

Magallanes' API is organized in two main modules: search engine and workflow composition.

### Search engine

The search engine module provides Google™-like methods for finding web resources using a scoring system based on the number of occurrences and relative word positions of matching hits. Currently it is endowed with AND/OR operators and regular expressions. The searching space defined by the resource metadata is easily expandable [see Additional file 1]. The algorithm initially searches for words similar to the keywords on the metadata descriptions. The similarity threshold can be setup as a configuration parameter [see Additional file 1]. If no hits occur, it becomes necessary to fall back on approximate expression matching. There are two widely used approaches for approximate expression matching: the Hamming distance [12], which compares strings of the same length and the Levenshtein distance [13], which compares two strings not necessarily having the same length, measuring by the minimum number of insertions, deletions, and substitutions of characters required to transform one string into another. Levenshtein distance is also known as the matching with *k *differences or errors. If the search does not generate hits, a "Did You Mean?" module in Magallanes pops up to aid the user. This module offers plausible alternatives to the user's query by computing the Levenshtein distance automatically (and letting the user influence the suggestions) to identify words similar to each keyword, and to estimate the distance using multiple keywords.

Magallanes uses a feedback module to continually learn and refine its discovery capabilities. Any client software using Magallanes is able to access this feedback module, which records user selections of resources associated with specific keywords. The module stores this information and records the 'feedback' value associated to the keyword-resource tuple (KR). This value is adjusted when the user selects another resource using the same keyword.

Selected KR tuples increase their *feedback *value (v) using the function v = v*α + (1-α), where α is a decay value [0-1 ranged] to slope the learning curve, and correspondingly all the remaining KR tuples with the same keyword decrement their value to v*α.

Results are ranked by combining the metadata matching information (id, name, description, documentation, etc.) with the feedback information when available. A *score *is computed as the average value of both. The first value is computed as the hits density in the matching space; in other words, the rate between the number of hits and text length. All density values are averaged and normalised to [0-1] to produce the metadata *score*. The *feedback *value for a given resource is the average of the KR tuple value for all the keywords used in the query.

Finally, Magallanes also allows the use of third-party discovery functionality. For instance, several repositories implement discovery strategies based on web service compatibility with a given data type (i.e., which services are able to process my data?). Intuitively, the consecutive application of this strategy can be exploited to create a sequence of compatible services that connect a given input with another target data type, in "pipeline" fashion. This motivates the next major area of functionality offered by Magallanes: the automatic arrangement of services to connect differing data types, including the management of user interactions to refine results.

### Automatic workflow composition

The Workflow Management consortium (WfMC) defines a workflow or workflow model as the complete or partial automation of a process in which information or tasks are passed from a participant to another according to a defined set of procedural rules.

Bioinformatics research can often benefit from connecting several applications in sequence to form a workflow (WF). Manual construction of WFs is complex and prone to error, particularly in bioinformatics where data comes in a multitude of formats. Combined with the difficulty of using distributed web services, composing a meaningful WF can present a challenge to life scientists.

Automatic workflow generation (also called automatic service composition) aims to automate the task of connecting independent services. Two services can be connected if the output of one is compatible with the input of the other. Therefore, the task of automatic workflow generation is to find the shortest non-redundant sequence of services, meaningful to the research, that match outputs with inputs to link the source to the target data type.

Workflow generation support can be either semi-automatic, interactively giving advice on suitable services for each step in workflow construction, or fully automatic, where the scientist only provides input and output data sets and the algorithm generates the complete workflow.

In simplest terms, the automatic WF-builder in Magallanes proceeds to identify all the services that produce a *target *data type as output. All the data types used as input for such services are used a target in the next step.

A well defined data type hierarchy will provide the required semantics to generate meaningful workflows.

A breath-first with pruning algorithm [14] speeds up the process of finding the shortest path from source to target (see Discussion section).

The following definitions are needed for formal statement of the algorithm: let D be the set of all the data types, and D* be the set of all the possible subsets of data types (that will be consumed by the functions H), thus D* = {d ⊆ D }.

Let T be the set of all the registered tools in the current repository and let P be all the tools I/O combinations (valid combinations D-T-D without inheritance). Therefore, P = {i, t, o: i, o ∈ D, t ∈ T | i is an input of t and o is an output of t }.

Here H^- ^: D → D* is a function defined to consume a given data type or any of the corresponding sub-data types as input: d_i _→ d = { dj ∈ D: d_j _is subtype of d_i _}.

As can be observed, H^-^: D* → D* extends the function to receive a set of data types to return all the subtypes of any of them D_i _→ d = { d_j _∈ D | ∃ d_h _∈ D_j _: d_j _is subtype of d_h _}.

The inverse function H^+^: D → D* defines the supertype d_*i *_→ d = { d_*j*_∈ D: d_*i *_is subtype of d_*j *_} and correspondingly H^+^: D* → D* extends the function to use collections of inputs: D_*i *_→ d = { d_*j *_∈ D | ∃ d_*h *_∈ D_*j *_: d_*h *_is subtype of d_*j *_}.

Using these definitions we can outline the algorithm:

Function Compass

   Input: source: D

      target: D

(1)   sources: ∅

(2)   unexplored: {target}

(3)   explored: ∅

(4)   depth [target]: 0

(5)   maxDepth: infinity

(6)   while unexplored != ∅

(7)      current: first of unexplored

(8)      unexplored = unexplored - current

(9)      explored = explored ∪ current

(10)         if depth [current] > maxDepth

(11)            end

(12)         if current ∈ H^+^(source)

(13)            sources = sources ∪ current

(14)            maxDepth: depth [current]

(15)            continue

(16)         ∀ p = (p_*i*_, p_*t*_, p_*o*_) ∈ P: p_*o *_= current

(17)            unexplored = unexplored ∪ p_*i*_

(18)            depth [p_*i*_]: depth [current] +1

(19)            suc [current] = suc [current] ∪ p_*i*_

Outputs:

sources: possible WF's input

suc [*dt*]: p ∈ P backtrack information from *dt *to a source.

When the *sources *set is empty means a partial solution was obtained; otherwise, a full solution has been reached. It is possible to modify the algorithm's behaviour to search for solutions other than the shortest by managing the *Depth *threshold parameter and by using *a-posteriori *refinement of the solution space by user interaction.

To illustrate the algorithm behaviour, a trivial example with a reduced set of data types (DT) and services registered in a repository will be used (see Table 1). The example consists of obtaining a set of amino acid sequences in Fasta format (FastaAAmult target DT) that are similar with a given an AASeq (source DT).

**Table 1:** Data types and services used in the example

**Input DT**	**Service**	**Output DT**
Object	getAASequence	AASeq
	getAASequenceCollection	AASeq
VirtualSeq		
GenericSeq	fromGenericSequenceCollectionToFasta	Fasta
	fromGenericSequenceToFasta	Fasta
	fromGenericToAASequence	AASeq
AASeq	runBlastp	BlastText
	runTblastn	BlastText
NNSeq	runBlastn	BlastText
	runBlastx	BlastText
	runTblastx	BlastText
TextPlain		
TextFormatted		
BlastText	getBestHitsFromBlast	Object
	getIDsFromBlast	Object
	parseMultipleAlignFromBLASTText	FastaAAmult
Fasta	fromFastaToAASequence	AASeq
	fromFastaToGenericSequence	GenericSeq
	runDisruptionPhysicalProperties	TextPlain
FastaAA	fromFASTAToAASequence	AASeq
	runPSIBlastpFromFASTA	BlastText
FastaAAmult	fromFASTAToAASequenceCollection	AASeq

Schematic representation of a reduced set of data types and associated services able to process these different types of data. Although tools names are descriptive, a long description is available as supplementary material [see Additional file 1].

The content of the variables during the execution of the algorithm evolves as follows (see Table 2): 'sources' will contain all the possible WF inputs; 'unexplored' is a list with the remaining DTs to analyse, in this case the target FastaAAmult DT and 'explored' is the list with the already analysed DT. The variable 'maxDepth' contains the depth of the shortest solution, and depth [<DT>] contains the specific values for each data type. Finally, suc [<DT>] is the set of services that produces the target from <DT> source in depth [<DT>] steps.

**Table 2:** Execution trace

**Step**	**Status**
Init	sources = ∅
	unexplored = { FastaAAmult }
	explored = ∅
	maxDepth = ∞
	depth [FastaAAmult] = 0
1	current = FastaAAmult
	sources = ∅
	unexplored = { BlastText }
	explored = { FastaAAmult }
	maxDepth = ∞
	depth [BlastText] = 1
	suc [BlastText] = {parseMultipleAlignFromBLASTText }
2	current = BlastText
	sources = ∅
	unexplored = { AASeq, NNSeq, FastaAA }
	explored = { FastaAAmult, BlastText }
	maxDepth = ∞
	depth [AASeq] = 2
	depth [NNSeq] = 2
	depth [FastaAA] = 2
	suc [AASeq] = {runBlastp, runTblastn}
	suc [NNSeq] = {runBlastn, runBlastx, runTBlastx}
	suc [FastaAA]={parseMultipleAlignFromBLASTText}
3	current = AASeq
	sources = { AASeq }
	explored = { FastaAAmult, BlastText, AASeq }
	maxDepth = 2
4	current = NNSeq
	sources = { AASeq }
	unexplored = { FastaAA }
	explored = { FastaAAmult, BlastText, AASeq, NNSeq }
	maxDepth = 2
5	current = FastaAA
	sources = { AASeq }
	unexplored = ∅
	explored = { FastaAAmult, BlastText, AASeq, NNSeq, FastaAA }
	maxDepth = 2

Tracing information showing the content of the main variables during the step-by-step algorithm execution.

In step 1, 'current' is assigned with 'FastaAAmult', this data type is removed from the 'unexplored' list and added to the 'explored' list. For each tool that returns the 'FastaAAmult' data type (in this case only 'parseMultipleAlignFromBlastText), add the inputs to the 'unexplored' list (BlastText) and set the depth of each input to depth [FastaAAmult]+1 (a new service is in the path). Finally, add the tool to the input data types' successors.

In Step 2, proceed as in step 1 by taking 'BlastText' from the 'unexplored' list as the 'current' and including 'AASeq', 'NNSeq' and 'FastaAA' to the 'unexplored' list, all of them with a depth value equal to 2.

In Step 3, 'current' takes 'AASeq' from the 'unexplored' list. Since 'AASeq', is the *source *data type, the algorithm adds 'AASeq' to the 'sources' list and removes it from the 'unexplored' list. Set maxDepth to the depth [AASeq] value (in this case 2).

In step 4, 'current' takes and moves 'NNseq' from the 'unexplored' to the 'explored' list. Since there is no tool that returns 'NNSeq' data type, go to the next step.

In step 5, proceed as in step 4 by selecting 'FastaAA' from the 'unexplored' list as the 'current' data type. The algorithm ends at this step because the 'unexplored' list is empty.

**Figure 1:**
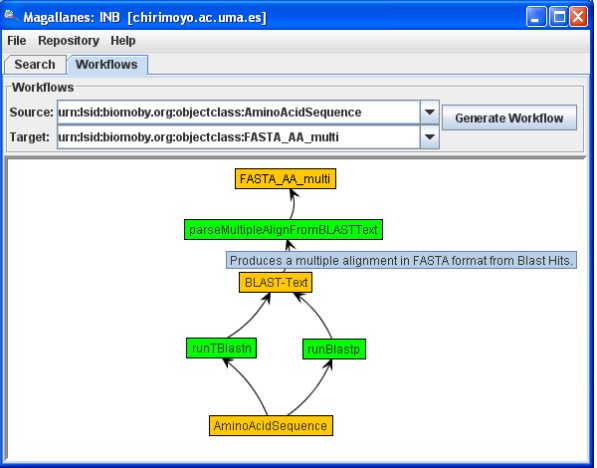
**Workflow example**. Proposed WF composition to obtain a set of sequences similar to a given amino acid sequence. Two alternative paths were identified and displayed to be edited and fine-tuned by user interaction.

The only possible workflow input is sources = {AASeq}. To built the graph (see Figure 1), WF sources are retrieved from sources and the successors can be recursively obtained from suc (i.e. suc [AASeq] = { runBlastp, runTblastn }. This means that the target data type (the solution) is closer when using AASeq to call runBlastp or runTblastn).

## Results

In this section, we describe the role and operation of Magallanes client. Magallanes' API functionality is available as a set of Java methods [see Additional file 1] that can be used by external clients in such a way that results can be used to invoke web services, recover data type descriptions, build up workflows, etc. Specific clients can be developed to fulfil services inter-operability, to enhance search engines, include the "*Did you mean?*" method, etc. [see Additional file 1]. It is noteworthy to observe that 'did you mean methods' has a long tradition of support of searching engines in the web environment, so what we claim is the novelty of the design, incorporation and utility of this type of strategies in the bioinformatics application domain.

**Figure 2:**
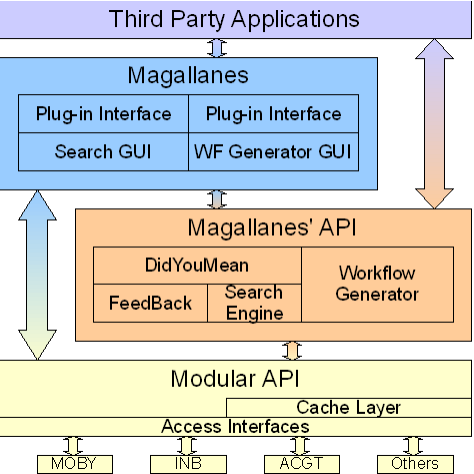
**Magallanes architectural scheme**. The bottom layer of the Magallanes' API library works over a standardised view of the diverse repositories and data models supplied by the modular API framework. Magallanes' API functionality is exposed to external clients as a powerful programmatic API organised in two main groups: searching and workflows methods.

Magallanes is a client with simple but powerful architecture for resource discovery and workflow composition (see Figure 2). As previously explained, the bottom layer of Magallanes' API supplies a uniform view of different data models by managing the uniform representation of resources from different repositories.

Magallanes, coded in Java, provides:

• integration of different repositories through the use of the modular API in such a way that discovery can be defined for multiple or individual repositories;

• the capability to extend the metadata discovery space to web links available in descriptive metadata;

• 'did you mean' assistance methods and user-profile learning capabilities;

• wide and extendable functionality to include new calling methods using the types of data available; and finally,

• several alternatives for user interaction, using the GUI library to directly built-up a desktop application, or developing a web-based tool by direct use of Magallanes' API; or as a discovery engine embedded in third-party applications.

Just like Magallanes' API, Magallanes client is organised into two main modules: searching and workflows. Both are GUI coded as Java Swing components [15] for querying and managing results, providing extra functionality such as service-data type compatibility.

The WF composition module is independent from the discovery engine, but the client can send data between the modules (e.g., set a data type as WF's input or output). The interactive GUI uses the JUNG framework [16] for graphing.

Magallanes is available as desktop application, Java Web Start, web page, web service implementation, or portlet in a Grid-Environment (currently, automatic workflow generation isn't available in Magallanes' web version). It can also be embedded in third-party applications like the jORCA client .

**Figure 3:**
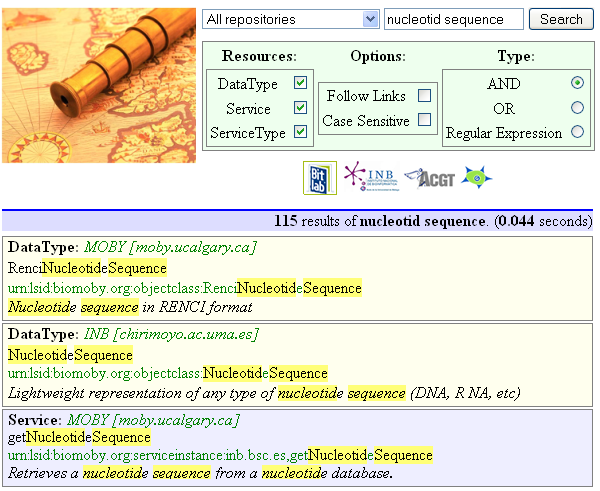
**Web-based implementation of Magallanes search engine**. Example of the web-based implementation of Magallanes engine using the default options for the "nucleotide sequence" query. Results are shown ranked by score, including the specific repository and resource. The web-search option follows web links specified as part of the service metadata.

Figure 3, depicts Magallanes' web interface using the key words 'nucleotide sequence' as a simple example. In a more elaborate query such as "How can I obtain a phylogeny for the gene I have?" the user could start by searching 'gene' (generating 512 hits), or 'phylog' (generating 62 hits--observe this is equivalent to phylog*) or finally, search for "gene phylog" which generates 14 hits. The second and third matching results correspond to 'Estimate phylogenies from protein (or nucleotide) sequence by unrooted parsimony'. It is interesting to note that the 'gene' keyword does not appear in the service description but in the web pages associated with the service. Magallanes follows the links in descriptions to also search for potential matches. This feature allows Magallanes to out-perform current search engines, and function as a discovery engine.

For a deeper exploration of Magallanes' capabilities, let us assume that we wish to obtain a multiple sequence alignment in Newick format using a given generic sequence as the starting point. The 'Did you mean?' module manages spelling mistakes (e.g. "seqeunc") and suggests a set of possible solutions (data types and services) related with the word 'sequence', among them the 'GenericSequence' data type that can be used as initial input into a WF. Note that GenericSequence is an example of a BioMoby specific data type that is likely unknown to the average user. The 'Did you mean?' module was able to inform the user about this data type. A second search using 'tree' keyword will identify "Newick_tree" that can be entered as target data type. Then the algorithm selects the "shortest" path, which could be extended by relaxing the *depth *threshold or by expanding one more level in a particular step.

In some research endeavours, a problem arises when only a partial solution can be found and there are no services to connect a given data type with the source data type by reverse analysis. Magallanes manages this situation by inserting a Black-Box service to complete the pathway, letting the user search manually for a solution.

### WF editing

Although WF editing is beyond the scope of Magallanes, the generated WF model can be examined by an expert user using the developed GUI, which has two switchable and synchronized graph layouts to provide alternative graph representations. The expert user understands how to analyse the WF model and, at the end of this stage, the model can be validated and accepted. In addition, before validating, the expert can review further feedback on the quality of the WF model derived by the quality of the individual services that make up the workflow. Some systems such as MOWServ [4] store information about service performance: CPU time, availability rate, frequency of use against alternative services, etc.

The resulting WF model can be stored in a SCUFL format for editing using the Taverna 1 [7] application. Final mapping with end-point services will occur in run-time.

## Discussion

### Scoring system: resource retrieval

The rationale of the scoring system used to rank resources is to combine the learning rate based on traditional KR voting systems with user's feedback. This enriches resource identification and adapts resources more rapidly to user needs. For instance, Magallanes stores feedback information using a file system--local in the case of desktop installations or on the server for web-based installations. In these different conditions, context sensitivity can be controlled by managing the learning rate. For instance, the typical scenario for desktop implementations is an individual user, thus a rapid learning rate is appropriate to accelerate adaptability to user preferences. However, a web-based application is designed to be used by several users, so a slower learning rate would improve stability and better reflect group behaviour.

### WF composition

Depth- and breath-first with pruning implementations were both evaluated for WF composition. Depth-first was able to identify the shortest solution by using an adaptive threshold. However, the repetitive exploration of the potential-solutions space is a challenge when efficient implementation is the goal.

Initial breath-first implementation drives a forward exploration from *source *to *target *data type with poor response times. The main reason for the excessive response time is related to the large number of services that uses a generic object as input; the typical root in the data type taxonomy system [see Additional file 1]. As result, object-input services always appear to be initially compatible.

However, a breath-first backward implementation from *target *to *source *data types produces good response times. Basically, this is because there are many more ways to consume a data type in current repositories than ways to produce data of a specific data type.

In graphical terms, forward connection is equivalent to the query, 'Which services can use a given DT?' This approach becomes expensive because of the large number of services that consume the root object (e.g. 'Object'). Backward compatibility asks 'Which services return a given DT?' Situations can arise when the list is empty since several DT are used as input by the services (but no services produce them); however, in general, the list is much shorter than in the forward approach.

Our breath-first algorithm is quicker than the deep-first one, although it consume more memory. To ensure that memory demand in breadth-first approach do not represents a problem we have tested the algorithm using the University of Calgary's BioMoby repository with around 786 services and 1655 data types registered. No memory problems arose.

### About the "Did you mean?" methodology

Levenshtein distance is used to identify similar words in the repository, producing a ranked list of possible solutions available to the client. This strategy ensures an up-to-date dictionary that is adapted to a specific repository (e.g., in different languages), but it becomes influenced by the quality of annotations in the repository (as when misspelled words on the repository cannot be detected).

The initial search approach is perfect matching which can produce an aesthetic situation: if a spelling mistake is made during the metadata resource annotation and the same mistake is used as query keyword, Magallanes will identify the mistake as the best solution and will suppress the "Did you mean?" module. However, this functionality (on/off methods) can be managed by the Magallanes' API.

Another discovery alternative is the use of dictionaries or ontologies to link related concepts, such as "FASTA → sequence → genome" providing semantic information that can be exploited by reasoning engines. However, a generic approach fits better for a broad range of applications, and also, the system is easily extendable to incorporate specific discovery mechanisms.

### Complex workflows (more than one input)

Without losing the advantages of generality, the described procedure enables users to discover alternative pipelines that connect source with target data types. However, some services need more than one input data type that, in turn, needs to be obtained via another pipelined branch of services. The current solution uses the iterative application of the algorithm for each of the needed branches and integrates them into a global solution [see Additional file 1].

### Service discovery

MOWServ [4] provides several alternatives for service discovery. The taxonomies for services and data types are presented in a tree which the user can browse and search. The search shows the number of hits and highlights the hits in yellow. The user must expand the trees to identify the hits. Furthermore, the trees always contain all resources, not only the matching hits, which make it difficult to find the hits. Searches are limited to full-string matching (allows for intermediate spaces). Additionally, services can be located based on the input data (similar to Seahawk and GBrowse). Recent additions to MOWServ allow users to search for services based on input data types, service type (keyword describing the semantics of the service) and output data types.

As mentioned before, Seahawk [5] analyses user data to determine the correct BioMoby data type. Based on this information, the application presents the user with available services grouped according to keywords (service types). There is no direct searching of service or data type descriptions.

Remora [6] displays available services based on the currently produced data type during workflow construction. Furthermore, the application provides search functionality that selects search terms based on direct or partial matches. Search terms must be exact and cannot be misspelled.

Taverna [7] has updated its search functionality in version 2.0. The application now allows direct and partial matching of nodes in the tree of available activities (BioMoby being one) showing also the number of matches. The tree is automatically filtered to show only matching results.

Taverna has a plug-in for FETA that allows the user to discover services based on name (partial or entire matches) or additional constraints where the user can select concepts from a list (task performed, data resource used, method used [algorithm etc.] and input/output data types). Concepts used in the search are taken from the myGrid ontology [17].

### Workflow generation

Magallanes performs automatic workflow composition, generating the entire sequence of services from start to finish datatypes. After the workflow has been generated, the user can select alternative paths. This strategy is less interactive than [10], but Magallanes was never designed to be a complete workflow editor. Instead, its focus was on service, data type and workflow discovery. Service and data type discovery allows clients to find the required data types and services using text searches in descriptions. Workflow discovery is not only supported in the obvious way, by treating workflows as services, but also by generating interesting workflows on-the-fly, allowing users to "discover" potentially interesting workflows and then export them to a fully-fledged workflow editor such as Taverna.

Magallanes' workflow generation is based on the hits generated in the search engine (see Magallanes' architecture section) where the user can choose to select a data type as source or target for the workflow generation algorithm.

As proof of concept we choose to test the workflows generation module to reproduce already published workflows like [18]. The workflow reported by Kerhornou and Guigó supports the clustering of co-regulated genes, producing as main result a hierarchical clustering in Newick format from a collection of DNA sequences.

Magallanes is able to find multiple alternative paths to solve that problem from a FASTA_NA_multi (a collection of nucleotide sequences in FASTA format) to produce a clustering in Newick format (Newick_text data type). The main path of that workflow (ignoring the image output) can be obtained making only four branch selections on Magallanes. This task only took a few minutes to complete with Magallanes, comparing to around two months needed for the manual elaboration of the same workflow (personal communication) [see Additional file 1].

## Conclusion

One of the most relevant research methods in bioinformatics is intensive use of distributed web-accessible resources. As a number of recent technical publications suggest, appropriate tools for resource discovery and for composition of complex workflows have become urgently needed. Both discovery and composition are the new paradigms to support data processing in massive genomics analysis. In this document, we have acknowledged those new working paradigms and proposed effective solutions.

The Magallanes software library supplies an integrated framework to develop powerful discovery engines that help researchers find web-services and associated data-types. The rationale for Magallanes' design has been efficiency and usability. There is consensus in the genomics research community that one of the biggest barriers to the integrated use of remote resources is difficulty of locating the appropriate resource. Several techniques have proposed to solve this problem, with varying degrees of success. Magallanes represents advancement in practical web-resource discovering tasks, regardless of application domain. Approximate keyword matching and user profiling have demonstrated the power of simple approaches similar to the most commonly used way to locate web pages--search engines.

A second important feature available in Magallanes is its capacity to build up workflows by automatic and efficient analysis of alternative pathways. These pathways go from an initial type of data to a desired output by using a set of available and compatible services. Rigorous evaluations of different algorithm implementations lead to an efficient breath-first pruning algorithm from target to source followed by a backtracking procedure.

The Magallanes client integrates different sources of resource metadata outperforming current client search capabilities. Moreover, the inclusion of indirect information from the available web page links usually embedded in description metadata extends the scope of discovery.

Various implementations of Magallanes client have been deployed to demonstrate the potential utility of the Magallanes' API. Different variations of the same client (web-based engines, desktop applications, etc.) demonstrate the versatility of the software library. Several of these clients are being used in real installations such as the National Institute of Bioinformatics (Spain) and ACGT-EU project, to exploit BioMoby-based repositories. Web services from the EBI are also among the available service catalogues.

Although many interesting improvements are already planned for Magallanes, the current approach is an important step in the integrated exploitation of web services, with user interaction and client usability in the application domain of bioinformatics.

## Availability and requirements

•Project name: Magallanes.

•Project home page: 

•Operating system(s): Platform independent.

•Programming language: Java.

•Other requirements: Java 6 or higher.

•License: free software.

•Any restrictions to use by non-academics: none.

## Authors' contributions

JR designed and programmed Magallanes. JK tested the application and helped with the manuscript. OTS conceived of the study, participated in its design and coordination and helped to draft the manuscript. All authors have read, participated in, and approved the final manuscript.

## Supplementary Material

Additional file 1 **Supplementary material**. Information about Magallanes' API, complex workflows composition, how to extend the search space, detailed algorithm trace, Did You Mean algorithm example, repositories' statistics and published workflows discovery. Click here for file
