# Supplementary material for: Magallanes: a web services discovery and automatic workflow composition tool
Source: BMC Bioinformatics. 2009 Oct 15;10:334. doi: 10.1186/1471-2105-10-334 (PMC2771019; doi:10.1186/1471-2105-10-334)
Supplement: Additional file 1 — Supplementary material. Information about Magallanes' API, complex workflows composition, how to extend the search space, detailed algorithm trace, Did You Mean algorithm example, repositories' statistics and published workflows discovery. [file 1471-2105-10-334-S1.PDF]

Supplementary Material

Magallanes: a web services discovery and automatic workflow composition tool

Javier Ríos[1], Johan Karlsson[1] and Oswaldo Trelles[1]
[1]Computer Architecture Department, University of Malaga, 29080, Spain

Contents

1 Extending search space 2
2 Detailed tracing information about algorithm execution 3
3 'Did you mean' approach 8
4 Word stemming 9
5 Create a Complex Workflow 9
6 Repository statistics 16
7 Magallanes' API 16
8 Adding new WSDL repositories to Magallanes 17
9 Magallanes as web service 17
10 Discovering published workflows 21
11 References 26

1 EXTENDING SEARCH SPACE

Magallanes has been developed using an object oriented methodology that makes easy the implementation of extensions that allows Magallanes to search over new resources like Mirrors, Users, Groups, Namespaces, etc.

All the searchable resources on Magallanes implements a common interface (Resource) that force all the resources to implement common methods for identify the resource (getId, getRepo), calc the number of hits (calcHits), compute the mark (calcMark) and add the new words to the dictionary (addWords).

The developer should implement those methods using the functionality provided by Magallanes to compute the number of hits, calc the mark or add the words to the dictionary.

For example, if you want to add the Users to the search space you must implement the next class:

```
public class User extends Resource {
	protected String id;
	protected String repo;
	protected String name;
	protected String department;
	protected String description;

	protected User(String id, String repo, String name, String department,
				String description) {
		super(EResourceType.User);
		this.id = id;
		this.repo = repo;
		this.name = repo;
		this.department = repo;
		this.description = repo;
		this.repo = repo;
	}

	public String getId() {
		return id;
	}

	public String getRepo() {
		return repo;
	}

	public double calcMark(SearchEngine se) {
		double mark = se.mark(name);
		mark += se.mark(department);
		mark += se.mark(description);

		mark /= 3;

		return mark;
	}

	protected int calcHits(SearchEngine se) {
		int res = se.hits(name);
		res += se.hits(department);
		res += se.hits(description);

		return res;
	}

	protected void addWords(SearchEngine se) {
		se.addWords(name);
		se.addWords(department);
		se.addWords(description);
	}
}
```

Once you have implemented this class you can search users like the other resources.

2 DETAILED TRACING INFORMATION ABOUT ALGORITHM EXECUTION

Algorithm

Function Compass
Input: source : D
target : D
$sources_0 := \emptyset$
$unexplored_0 := \{target\}$
$explored_0 := \emptyset$
depth[target] := 0
maxDepth := infinity
while $unexplored_i \mathrel{!}= \emptyset$
  current := $unexplored_i[0]$
  $unexplored_{i+1} = unexplored_i$ - current
  $explored_{i+1} = explored_i \cup$ current
  if depth[current] > maxDepth
    end
  if current $\in H^+$(source)
    $sources_{i+1} = sources_i \cup$ current
    maxDepth := depth[current]
    continue
  $\forall$ p = (pi, pt, po) $\in$ P : po = current
    $unexplored_{i+1} = unexplored_i \cup$ pi
    depth[pi] := depth[current] +1
    suc_{i+1}[current] = suc_i[current] $\cup$ p

Outputs:
$sources_n$: possible WF's input
suc_n: p $\in$ P backtrack information to the target

Table SP1.- Full schematic representation of a reduced set of datatypes and services able to process these different type of data.

**DataType**	**Service**	**Output datatype**	**Service description**
Object	GetAASeq	AASeq	Sequence DB retrieval using a valid identifier
	GetAASeqColl	AASeq	Sequence DB retrieval using a set of valid identifiers
VirtualSeq			
GenericSeq	fromGenericSeqCollToFasta	Fasta	Convert a collection of sequences to Fasta format.
	fromGenericSeqToFasta	Fasta	Convert a sequence to Fasta format.
	fromGenericToAASeq	AASeq	Convert a generic sequence into an AASeq object
AASeq	runBlastp	BLAST-Text	Search protein database using a protein query
	runTblastn	BLAST-Text	Search translated nucleotide database using a protein query
NNSeq	runBlastn	BLAST-Text	Search a nucleotide database using a nucleotide query
	runBlastx	BLAST-Text	Search protein database using a translated nucleotide query
	runTblastx	BLAST-Text	Search translated nucleotide database using the six-frames translated nucleotide query

TextPlain			
TextFormatted			
BLAST-Text	getBestHitsFromBlast	Object	Get the best hits from a Blast report.
	getIDsFromBlast	Object	Parses a Blastp output text obtaining a list of objects with the identifiers and namespaces that appears in it with a identitie value bigger or equal to the provided one.
	parseMultipleAlignFromBlast	FASTA_AA_multi	Produces a multiple alignment in FASTA format from Blast Hits.
Fasta	fromFastaToAASeq	AASeq	Converts amino acid FASTA sequence into an amino acid sequence.
	fromFastaToGenericSeq	GenericSeq	Converts a sequence in Fasta format into a GenericSeq object.
	runDisruptionPhysicalProperties	TextPlain	This web service uns the following physical properties for a given dna sequence: Base-pair stacking, Duplex disruption free energy, Duplex stability free energy, Stacking energy
Fasta_AA	fromFASTAToAASeq	AASeq	Parses a fats formated sequence into a AASeq standard object
	runPSIBlastpFromFASTA	BLAST-Text	Perform 2 iterations of protein PSI Blast against non-redundant database with standard settings.
Fasta_AA_multi	fromFASTAToAASeqColl	AASeq	Converts amino acid FASTA sequences into a collection of amino acid sequences.

Execution example and tracing: **From AASeq to Fasta_AA_multi**

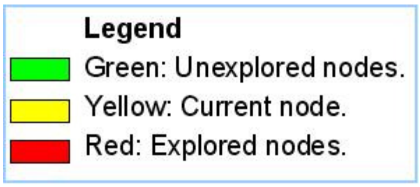

Using the set of datatypes and services described in Table SP1, compose a WF to get a set of amino acid sequence in Fasta format similar with a given an amino acid sequence.

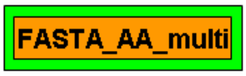	[Step: Init]<br>Initialize the unexplored nodes with 'FASTA_AA_multi', the target DataType.
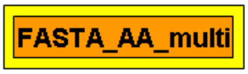	[Step: 1]<br>Select 'FASTA_AA_multi' as the current node from the unexplored list.
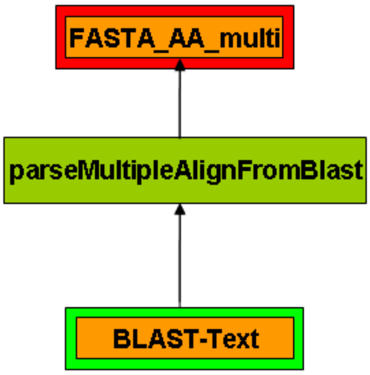	[Step: 1]<br>Add 'FASTA_AA_multi' to the explored list.<br><br>Only 'parseMultipleAlignFromBlast' returns an object of type 'FASTA_AA_multi'.<br><br>'BLAST-Text' is an input of 'parseMultipleAlign-FromBlast', add it to the unexplored list.<br><br>Link 'FASTA_AA_multi' with 'BLAST-Text' adding 'FASTA_AA_multi' to the successors list of 'BLAST-Text'.

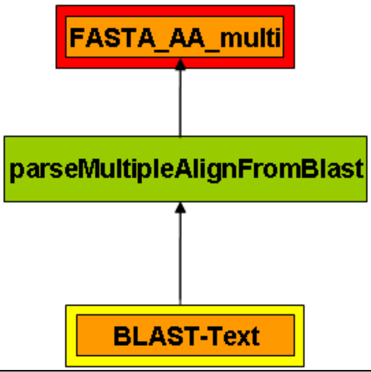

[Step: 2]
Select ‘BLAST-Text’ as the current node from the unexplored list.

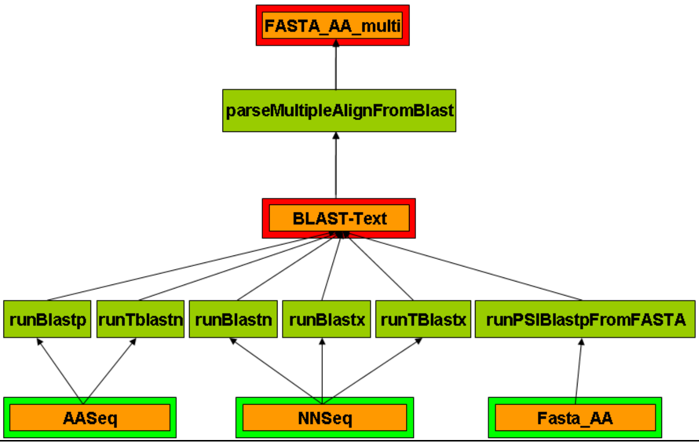

[Step: 2]
Add ‘BLAST-Text’ to the explored list.

There are multiple tools that returns objects of type ‘BLAST-Text’.

For each tool that returns a ‘BLAST-Text’ we should:

- Add all the inputs of those tools to the unexplored list.
- Add ‘BLAST-Text’ to the successors list of all the inputs of those tools.

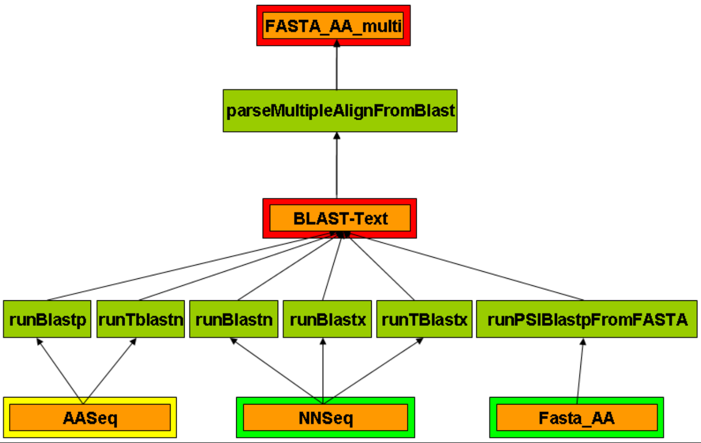

[Step: 3]
Select ‘AASeq’ as the current node from the unexplored list.

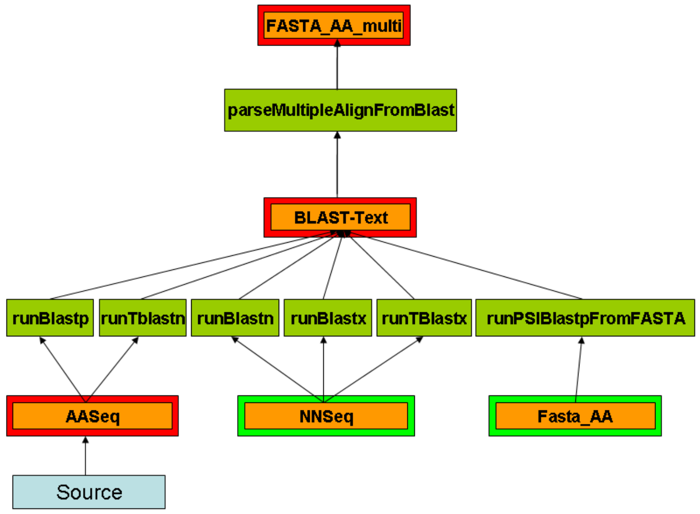

[Step: 3]
Add ‘AASeq’ to the explored list.

As ‘AASeq’ is the source datatype we will add it to the possible sources list.

We have found a solution at deep 2 (number of services). We set the max deep to 2.

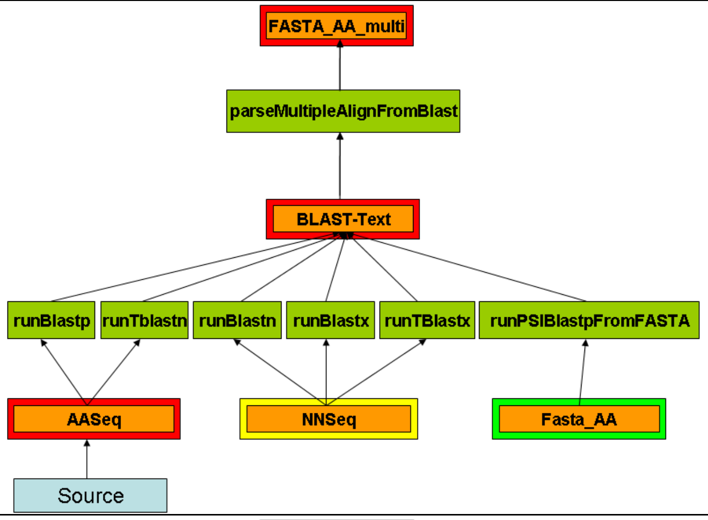

[Step: 4]
Select ‘NNSeq’ as the current node from the unexplored list.

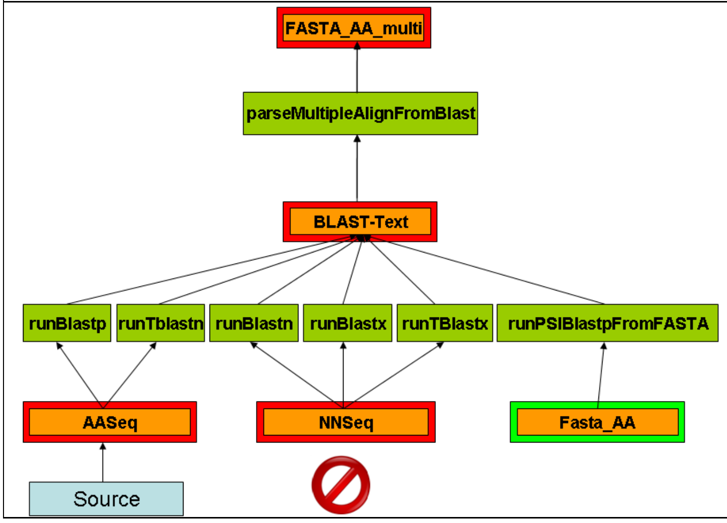

[Step: 4]
Add ‘NNSeq’ to the explored list.

‘NNSeq’ is at deep 4, all the successors of ‘NNSeq’ are deeper then we will ignore them.

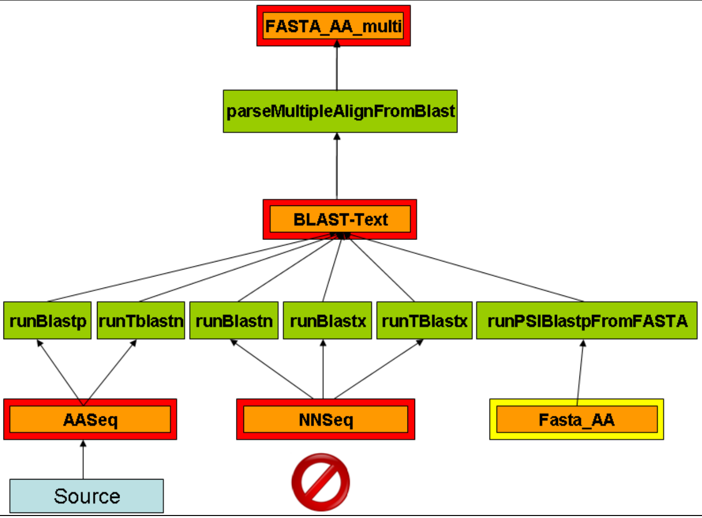

[Step: 5]
Select ‘FASTA_AA’ as the current node from the unexplored list.

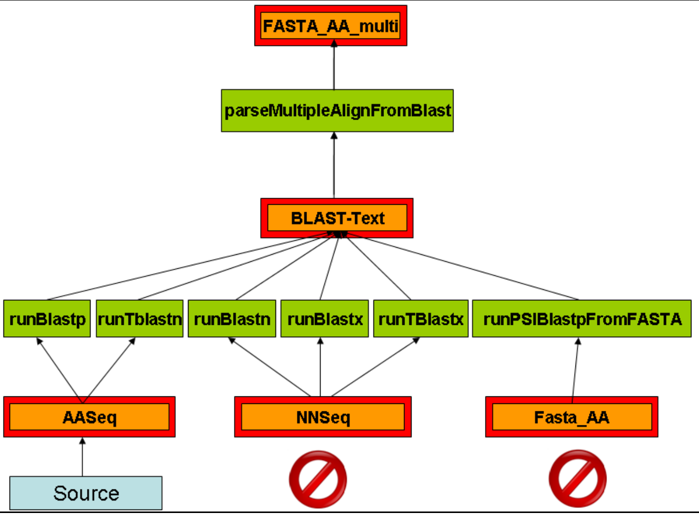

[Step: 5]
Add ‘FASTA_AA’ to the explored list.

‘FASTA_AA’ is at deep 4, all the successors of ‘FASTA_AA’ are deeper then we will ignore them.

The algorithm finish because the unexplored list is empty.

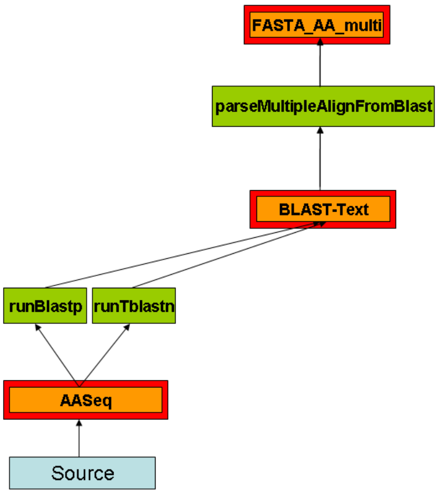

[Step: solution]
To reconstruct the solution, we will get the datatypes from the possible sources list and get it’s successors to obtain the resulted workflow.

3 ‘DID YOU MEAN’ APPROACH

Magallanes includes a DidYouMean module that tries to fix the user query when there are misspelling keywords.

The DidYouMean module uses a dictionary provided by the search engine that includes all the search space’s words and find the most probable combination of keywords that are too close to the user input and returns almost one result.

For example, if the user insert “aminoacid seluence” on the search box. The DidYouMean module will generate a list for each keyword with all the dictionary words ordered by the similarity with the user input (using the Levenstein distance).

aminoacid = { aminoacid, amino, annotated, acid, approach, … }
seluence = { sequence, sequences, equence, select, service, … }

The first DidYouMean suggestion will be generated taken the first element of each list resulting “aminoacid sequence” that is the correct one. If the user wants another suggestion it will be calculated on-demand taking the next best ranked word of one list and combining it with all the suggestions generated before.

If the user rejects all the suggestions the DidYouMean module will generate it as follow:

At first step we will have the suggestion’s buffer empty and we will get the first element of each list as suggestion.

Suggestion: “aminoacid sequence”

Used: “aminoacid sequence”
Buffer: <Empty>

If the user wants another suggestion, the last one will be added to the used list and if the buffer is empty it will be filled taking one word from a list of probable words and combining it with all the used suggestions.

The selected word is “amino”

Suggestion: “amino sequence”
Used: “aminoacid sequence” “amino sequence”
Buffer: <Empty>

At the next step “sequences” will be selected from the words list producing more suggestions:

Suggestion: “aminoacid sequences”
Used: “aminoacid sequence” “amino sequence” “aminoacid sequence”
Buffer: “amino sequences”

At the next step we have a suggestion on the buffer, then we don’t need to calculate another one:

Suggestion: “amino sequences”
Used: “aminoacid sequence” “amino sequence” “aminoacid sequence” “amino sequences”
Buffer: <Empty>

If the user wants another suggestion “annotated” will be selected as the new keyword:

Suggestion: “annotated sequence”
Used: “aminoacid sequence” “amino sequence” “aminoacid sequence” “amino sequences”
Buffer: “annotated sequences”

The DidYouMean module will be calculating suggestions on-demand as described until the user accepts one suggestion.

4 WORD STEMMING

About the use of a word stemming algorithm we analyzed two aspects:

a) How to obtain the similar words:
   i) Using a word stemming algorithm (a public available version from 'Porter algorithm' was plugged into Magallanes for testing purposes).
   ii) Searching similar words on the dictionary (automatically generated from the search space). The similarity level could be configured on the configuration file as a percent value.

b) How to manage the search results:
   i) Include the related words automatically into the user's query relaxing the search.
   ii) Add a button to manually broaden the search when the user wants to obtain more results.

The main results were:

- Stemming algorithms are based on suffix stripping searching for the root of the words, which in turn can introduce several errors[1] since behaves strongly related to the base language (e.g. English), and do not perform well for technical words. On the other hand we can obtain the related words by searching similar terms on the dictionary using the same current algorithm in Magallanes regardless of the language used to describe the associated metadata.
- The similarity is calculated during the dictionary generation –once for the used source of metadata- without time penalty in the performance of the search engine. But stemming algorithms are more expensive since they must examine a great many endings.

Based on this analysis the alternatives we decided:

- Getting the related words by searching similar terms on the dictionary.
- Broaden the search always automatically relaxing the user's query.
- The similarity threshold can be setup in the configuration file.

5 CREATE A COMPLEX WORKFLOW

Search using Magallanes the minimum workflow between AminoAcidSequence and FunCUT_Annotation_XML.

1 http://wwwcs.uni-paderborn.de/cs/ag-klbue/en/workshops/tir-04/proceedings/yamout04-further-enhancement-porter-stemming.pdf

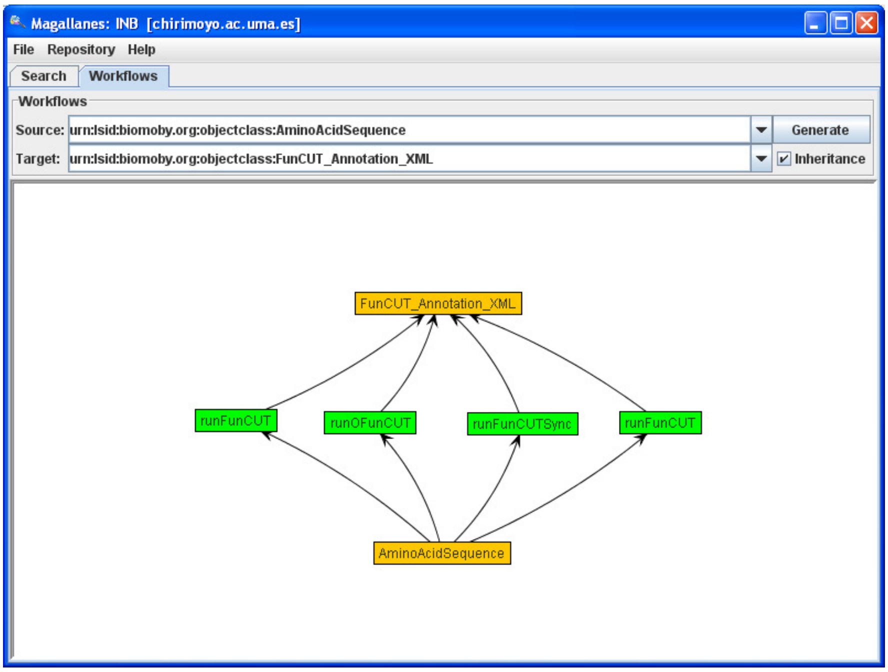

We have four alternatives to go from AminoAcidSequence to FunCUT_Annotation_XML. We will select runOFunCUT (double clicking over it) and export the workflow on Scufl format (right click and select Export).

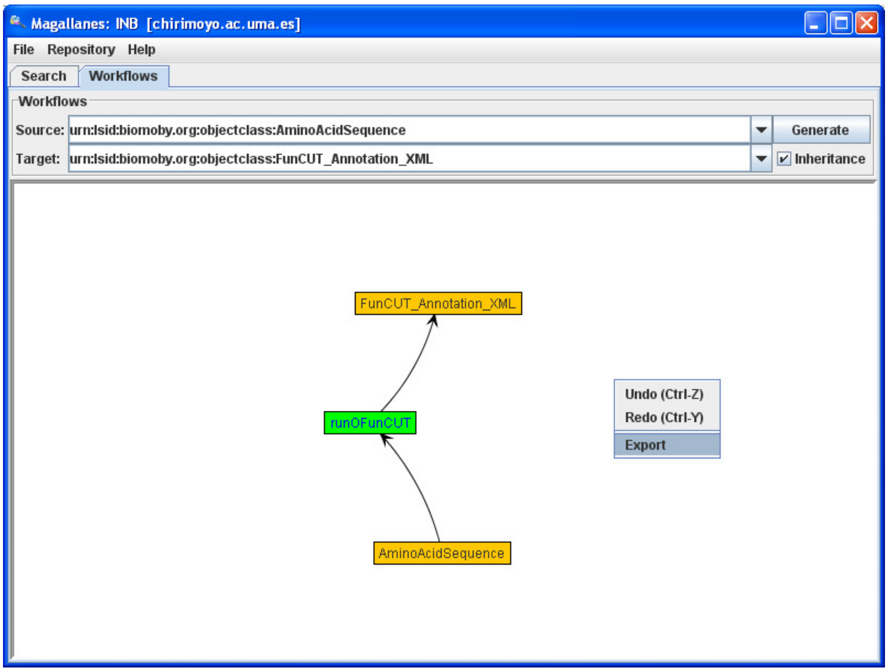

Now, open the workflow with Taverna and configure the graph to show “All ports” and “Top to bottom”.

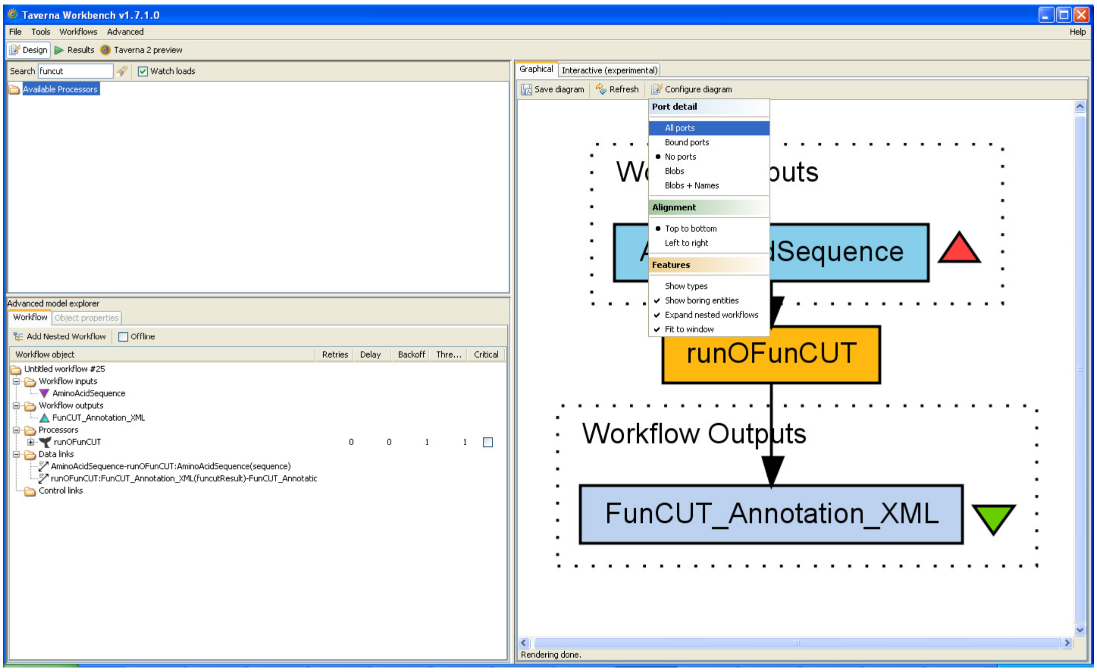

Now we need some more inputs to execute the runOFunCUT (for example Ncut_Clusters). We will use Magallanes again to search a workflow from our AminoAcidSequence to the Ncut_Clusters and export it to Scufl.

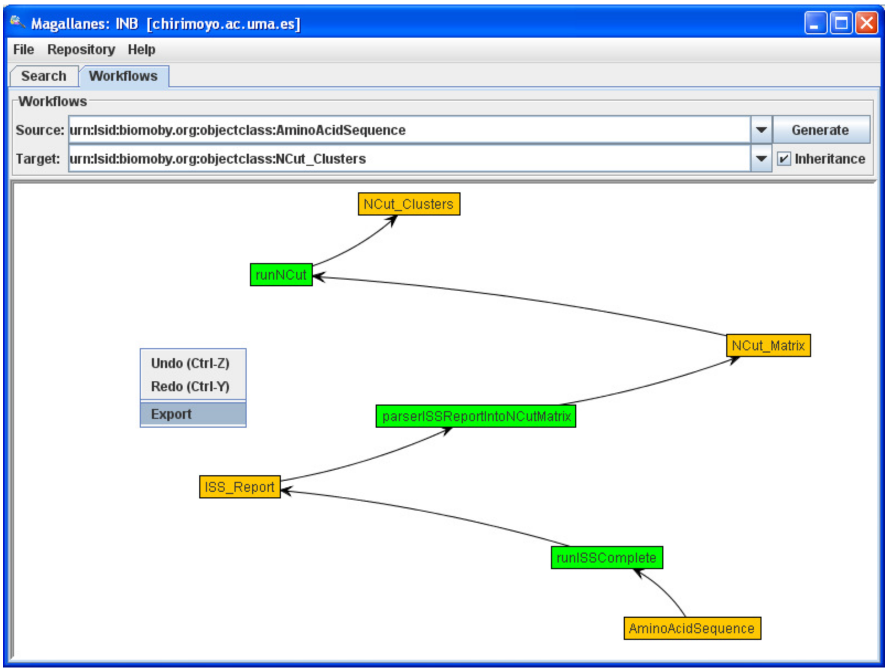

Add the new Workflow as Nested Workflow on Taverna.

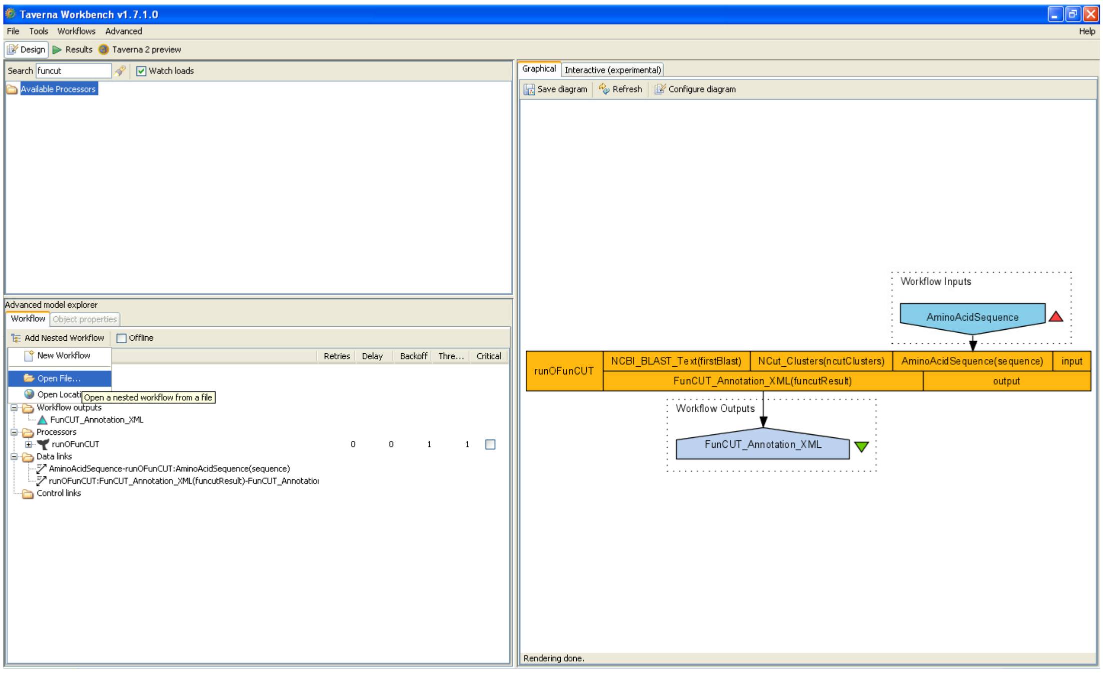

Link the nested workflow's output (NCut_Clusters) with our workflow as input of runOFunCUT.

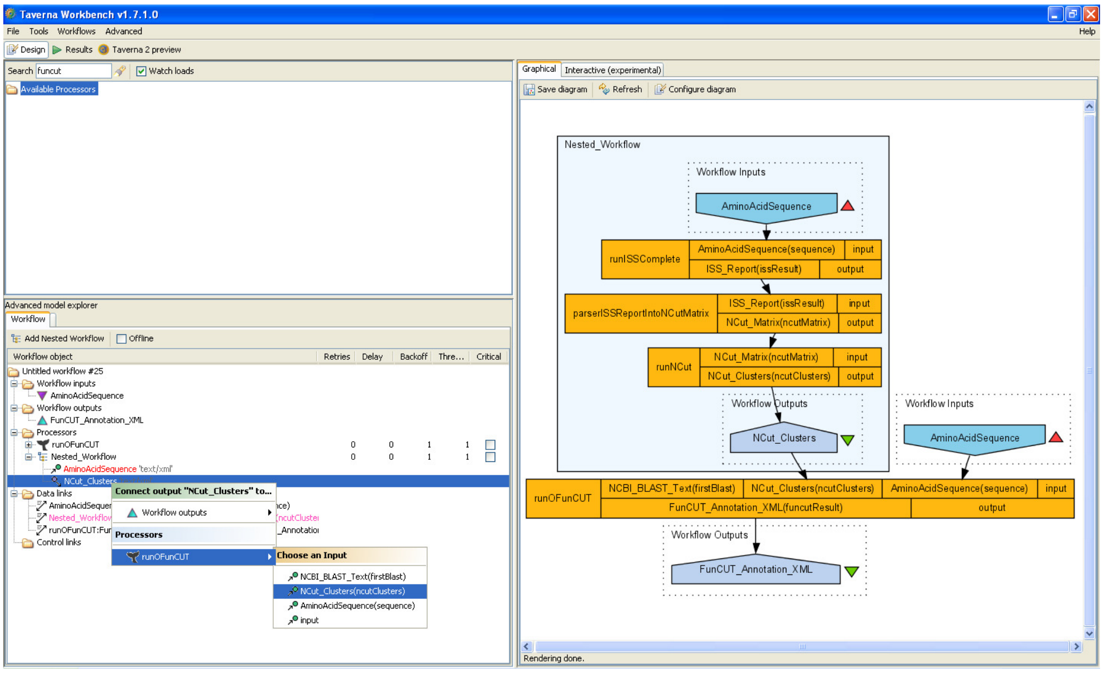

Link our AminoAcidSequence (workflows input) with the Nested Workflow's input.

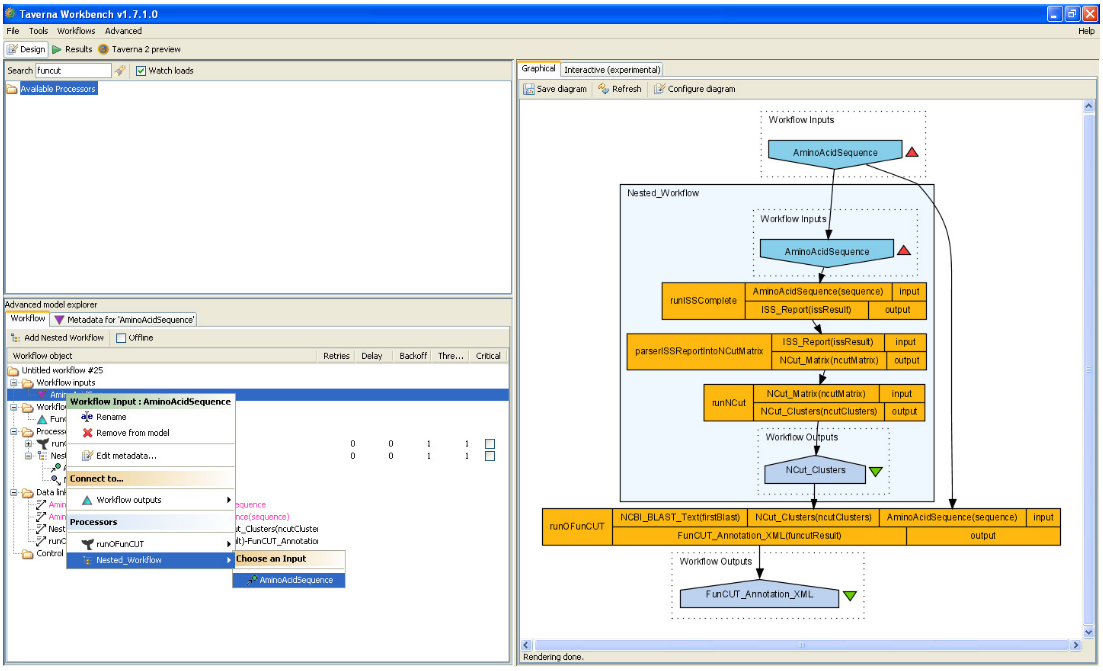

Search with Magallanes the path to go from AminoAcidSequence to NBI_BLAST_Text and export it to Scufl.

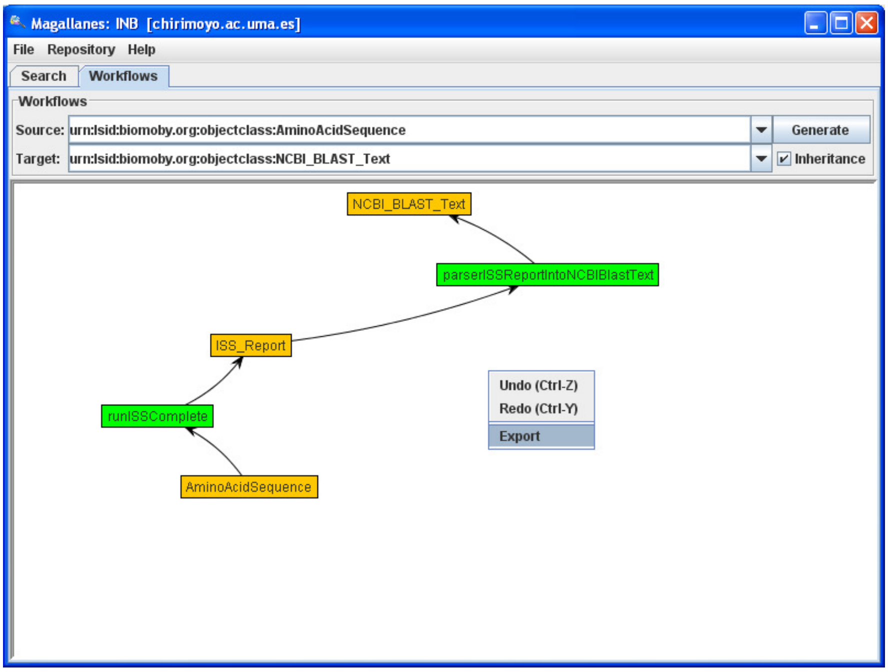

Load the Workflow as nested workflow in taverna.

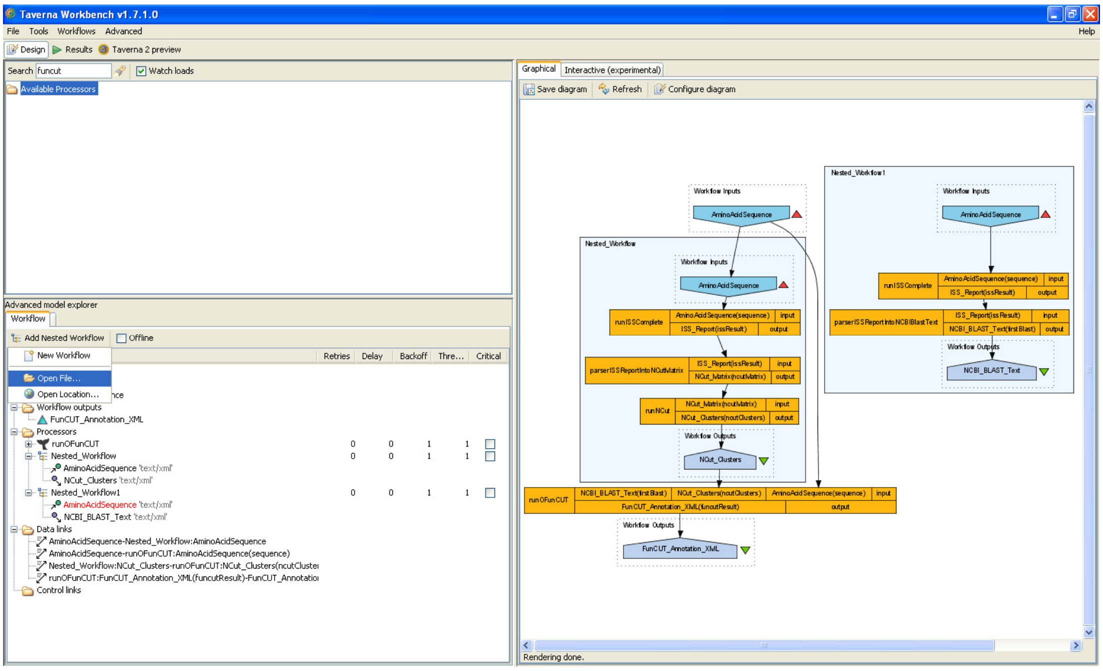

Link the nested workflow's output (NCBI_BLAST_Text) with our workflow as input of runOFunCUT.

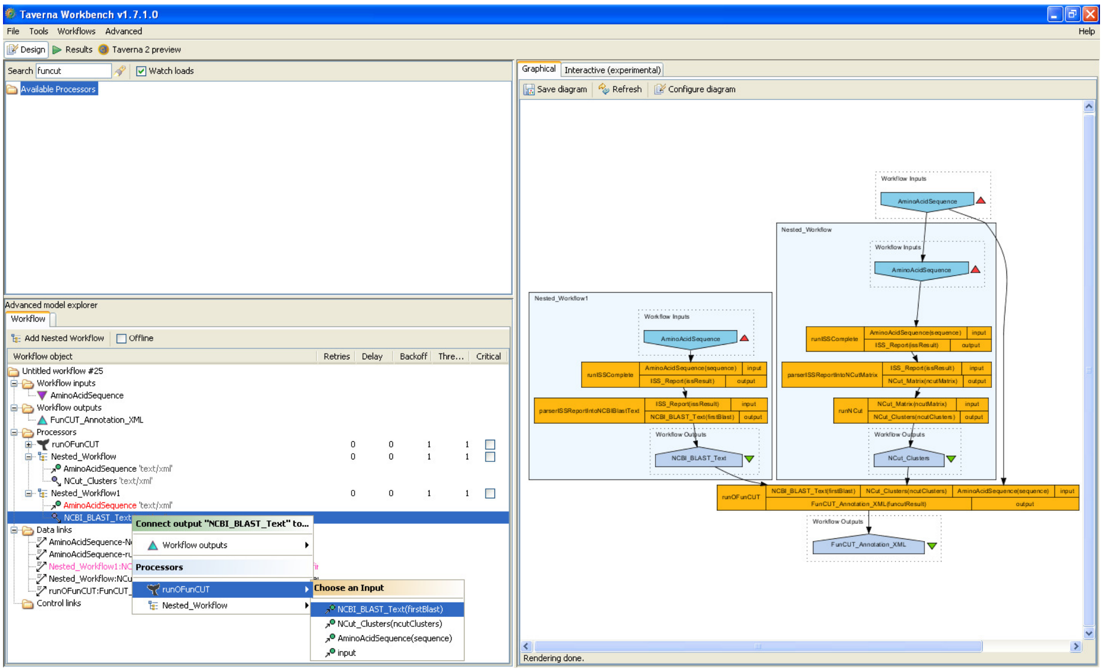

Link the Nested Workflow's input (AminoAcidSequence) with the nested Workflow's input.

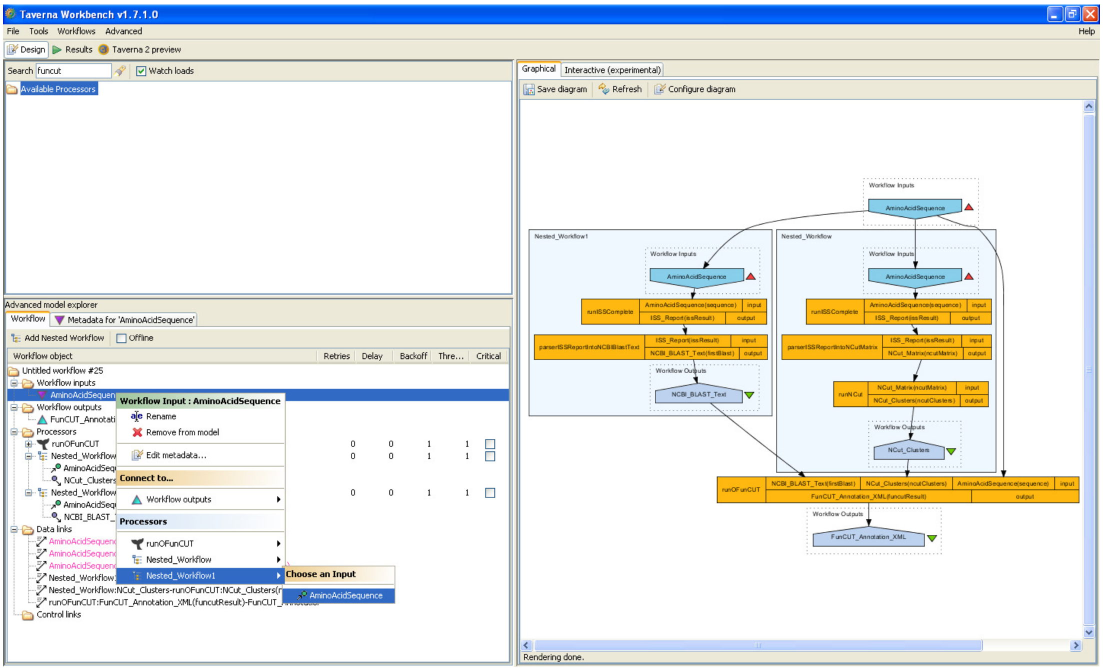

Now we have a full workflow. As you can see there are some common parts on the workflow, when you have some practice with the workflow generation you will be able to simplify and optimize the automatic generated workflows.

6 REPOSITORY STATISTICS

We have then two alternatives to perform the search:

1. Go forward (from source to target): is equivalent to query us on each step “What services are able to process my ‘current’ data type?”
2. Go backward (from target to source): is equivalent to query us on each step “What services are able to produce my ‘current’ data type?”

We can observe on the next table that there are a lot of services on the repositories used by Magallanes that the mean number of services that accept a concrete data type is grater than the number of services that produces one. This is really our branching factor when we are performing the search.

	Chirimoyo (UMA)		Moby-Dev (INB)		Moby-Canada	
DataTypes	181		296		786	
Tools	188		516		1655	
Tools with Object as Input	38		124		745	
Tools with multiple Outputs	4		17		116	
Inheritance	Yes	No	Yes	No	Yes	No
Tools for use DT as Input (Mean)	46,38	2,13	142,45	3,78	760,15	3,98
Tools for get a DT as Input (Mean)	3,95	1,04	6,94	1,79	6,74	2,24
DataTypes used only as Input	83	19	111	18	448	33
DataTypes used only as Output	0	50	0	93	0	169
Unused DataTypes	0	84	0	118	0	454

The space and time complexities of the breath-first is $O(b^d)$, with ‘b’ as the graph’s branching factor and ‘d’ as the solution’s deep. Then, we can save space and time if we do a backward search.

7 MAGALLANES’ API

The classes "SearchGUI" and "ResultLister" provide access to the application GUI and the "SearchEngine" class is used for low-level access (without graphical interface).

Java Class	Method	Input	Output	Description
SearchGUI	SearchGUI	JPanel panel	<<constructor>	Create a searcher instance in jpanel.
	addResource-Menu	EResourceType type<br>ResourceMenuOption option	-	Add a new option to the Results context menu.
	addResultListener	ResultListener listener	-	Add a listener on user selection over results.
WorkflowPanel	WorkflowPanel	JPanel panel	<<constructor>>	Create a workflow instance in JPanel.
ResourceListener	resourceSelected	DataType dataType	-	Function called when a DataType 'dataType' is selected.
	resourceSelected	Service service	-	Function called when the Service 'service' is selected.
	resourceSelected	ServiceType serviceType	-	Function called when a ServiceType ‘servicetype’ is selected.
SearchEngine	SearchEngine	List<Resource> source<br>String query<br>boolean caseSensitive<br>Boolean webSearch<br>EType type	<<constructor>>	Create a search engine for the EType 'type' on 'souce' with query 'query' and caseSensitive constrain.
	search	-	List<Resource>	Return back searching results.
	didYouMean	-	DidYouMean	Returns an object with similar to input keywords.

8 ADDING NEW WSDL REPOSITORIES TO MAGALLANES

There are currently two ways to add new repositories WSDL to Magallanes:

i) The easiest is to add the WSDL files to the 'wsdl' folder on the Magallanes installation directory (if you are using the JNLP version it will be '%HOME%/.magallanes/wsdl'). The services will appear in the 'WSDL [local]' repository.

ii) Add a new repository:
   (1) Copy one of the EBI or WABI configuration files (there are on the 'conf' folder) and edit it changing the WSDL's URLs.
   (2) Add the new repo to the 'Magallanes.conf' file *(<Repo name="WSDL [MyRepo]" file= "conf/api.wsdl.myrepo.cache.conf" />)*.

After restart Magallanes you will be able to search over your WSDL services.

9 MAGALLANES AS WEB SERVICE

Magallanes has been implemented also as a web service that can be easily used from external applications.

The WSDL is in: http://mango.ac.uma.es/magallanesWS/services/Magallanes?wsdl

To execute the operations from a standard web browser you can use a generic SOAP client (Membrane SOA Registry: http://www.service-repository.com/client/start).

This web service has 3 operations including all the Magallanes functionality.

Search:

This operation performs a search using the Magallanes' search engine.

Operation header:
SearchData search(boolean searchDataTypes, boolean searchTools, boolean searchFunctionalCategories, String pattern, boolean caseSensitive, boolean webSearch, String type, int maxDidYouMean)

Request example:

```
<search>
 <searchDataTypes>true</searchDataTypes>
 <searchTools>true</searchTools>
 <searchFunctionalCategories>true</searchFunctionalCategories>
 <pattern>gene expression</pattern>
 <caseSensitive>true</caseSensitive>
 <webSearch>true</webSearch>
 <type>AND</type>
 <maxDidYouMean>10</maxDidYouMean>
</search>
```

This example search the pattern "gene expression" on DataTypes, Tools and FunctionalCategories, with case sensitive, following the links (the links are in the Tool's long description) to increase the search space, using AND relation if there are more than one words on the pattern and returns 10 suggestions using the did-you-mean module.

The response XML:

```
<searchReturn>
   <didYouMean>geneexpression</didYouMean>
   <didYouMean>genesexpression</didYouMean>
   <didYouMean>geneexpressed</didYouMean>
   <didYouMean>genesexpressed</didYouMean>
   <didYouMean>gensexpression</didYouMean>
```

```
  <didYouMean>gensexpressed</didYouMean>
  <didYouMean>geneexpressded</didYouMean>
  <didYouMean>genesexpressded</didYouMean>
  <didYouMean>gensexpressded</didYouMean>
  <didYouMean>geneidexpression</didYouMean>
  <results>
    <dataTypeData>
      <creationTime>1970-01-01T00:00:00.000Z</creationTime>
      <description>Micro-array data in text format representing gene expression patterns. Each line must have the gene name and the expression values and the expression values for each condition. All the data items must be separated by tabulators</description>
      <id>urn:lsid:biomoby.org:objectclass:MicroArrayData_Text</id>
      <name>MicroArrayData_Text</name>
      <version>0.0</version>
    </dataTypeData>
    <functionalCategoryData xsi:nil="true" />
    <toolData xsi:nil="true" />
    <type>DataType</type>
  </results>
  <results>
    <dataTypeData xsi:nil="true" />
    <functionalCategoryData xsi:nil="true" />
    <toolData>
      <authorEMail>jtarraga@cipf.es</authorEMail>
      <authorName>Joaquin Tarraga</authorName>
      <authorityEMail>jtarraga@cipf.es</authorityEMail>
      <authorityName>bioinfo.cipf.es</authorityName>
      <creationTime>1970-01-01T00:00:00.000Z</creationTime>
      <description>Hierarchical clustering application for gene expression patterns using different conditions, methods and distances</description>
      <doc><?xml version="1.0" encoding="UTF-8"?> <help> <title>Hierarchical clustering for DNA array data</title> <paragraph>The UPGMA is an agglomerative hierarchical method (Sneath and Sokal, 1973), which is probably the most popular among the clustering algorithms in the field of microarrays. It is not the more accurate among the methods but is really extensively used (Dhaeseler, 2005). Hierarchical clustering starts by calculating the all-to-all distance matrix. The two closest patterns are merged and the all-against-all distance matrix is calculated again but using the new cluster instead of the two merged patterns. This process is repeated until the complete dendrogram is built.</paragraph> <title>More information</title> <link>http://gepas.bioinfo.cipf.es/cgi-bin/tutoX?c=clustering/clustering.config</link> </help></doc>
      <id>urn:lsid:biomoby.org:serviceinstance:bioinfo.cipf.es,runHierarchicalClustering</id>
      <name>runHierarchicalClustering</name>
      <version>1.0</version>
      <wsdl xsi:nil="true" />
    </toolData>
    <type>Tool</type>
  </results>
  ...
<searchReturn>
```


As you can see, the response has a list with the "did-you-mean?" suggestions and the list of results.
Each result can have a DataType, a Tool or a FunctionalCategory (only one of them) and the field "type" will show you the result's type.

Feedback:

This operation increment the feedback value of the resource with id ‘id’ for the keywords contained on ‘query’.

Operation header:

void feedBack(String id, String query)

Request example:

```
<feedBack>
  <id>urn:lsid:biomoby.org:objectclass:ClusterProfile_Text</id>
  <query>gene expression</query>
</feedBack>
```

This operation will be used to annotate that the resource with the id "urn:lsid:biomoby.org:objectclass:ClusterProfile_Text" as having been "selected by the user using the search query 'gene expression'". The next time the same query is used, that resource will be ranked higher.

Workflow:

This operation retrieves all the possible shortest paths from the ‘source’ to the ‘target’ DataType.

Operation header:
WorkflowData workflow(String source, String target, boolean inherit)

Request example:

```
<workflow>
  <source>urn:lsid:biomoby.org:objectclass:GenericSequence</source>
  <target>urn:lsid:biomoby.org:objectclass:GFF</target>
  <inherit>true</inherit>
</workflow>
```

This example will search a workflow starting from a ‘GenericSequence’ and returning a ‘GFF’ object using inheritance.

The response XML:

```
<workflowReturn>
  <dataTypes>
    <creationTime>2008-05-20T07:29:11.000Z</creationTime>
    <description>Generic Feature Format (GFF) text report. GFF is a format for describing genes or any other features
    associated with DNA, RNA and Protein sequences</description>
    <id>urn:lsid:biomoby.org:objectclass:GFF</id>
    <name>GFF</name>
    <version>1.0</version>
  </dataTypes>
  <dataTypes>
    <creationTime>2008-10-09T13:19:24.000Z</creationTime>
    <description>This is a baseclass for sequences</description>
    <id>urn:lsid:biomoby.org:objectclass:GenericSequence</id>
    <name>GenericSequence</name>
    <version>1.0</version>
  </dataTypes>
...
  <edges>
    <source>urn:lsid:biomoby.org:serviceinstance:genome.imim.es,parseMotifMatricesFromMEME;parseMotifMatricesF
    romMEME;COLLECTION;0;motif_weight_matrices</source>
    <target>urn:lsid:biomoby.org:serviceinstance:genome.imim.es,runMatScanGFFCollectionVsInputMatrices;runMatSca
    nGFFCollectionVsInputMatrices;COLLECTION;1;motif_weight_matrices</target>
  </edges>
  <edges>
    <source>urn:lsid:biomoby.org:serviceinstance:genome.imim.es,runMemeText;runMemeText;SIMPLE;0;meme_predict
    ions</source>
    <target>urn:lsid:biomoby.org:serviceinstance:genome.imim.es,parseMotifMatricesFromMEME;parseMotifMatricesFr
    omMEME;SIMPLE;1;meme_predictions</target>
```

</edges>
<full>true</full>
<inputs>urn:lsid:biomoby.org:serviceinstance:genome.imim.es,runMemeText;runMemeText;COLLECTION;1;sequences
</inputs>
<operations>
<description>Analyzes a set of protein or DNA sequences for similarities among them and produces a description (motif) for each pattern it discovers. The results are returned in MEME text format</description>
<id>urn:lsid:biomoby.org:serviceinstance:genome.imim.es,runMemeText;runMemeText</id>
<name>runMemeText</name>
<toolId>urn:lsid:biomoby.org:serviceinstance:genome.imim.es,runMemeText</toolId>
</operations>
<operations>
<description>Analyzes a collection of DNA sequences for putative motifs (transcription or splicing factor binding sites) and reports them in GFF format. The collection of motifs is given by the user as a set of Position Weight Matrices (PWMs)</description>
<id>urn:lsid:biomoby.org:serviceinstance:genome.imim.es,runMatScanGFFCollectionVsInputMatrices;runMatScanGFFCollectionVsInputMatrices</id>
<name>runMatScanGFFCollectionVsInputMatrices</name>
<toolId>urn:lsid:biomoby.org:serviceinstance:genome.imim.es,runMatScanGFFCollectionVsInputMatrices</toolId>
</operations>
...
<outputs>urn:lsid:biomoby.org:serviceinstance:genome.imim.es,runMatScanGFFCollectionVsInputMatrices;runMatScanGFFCollectionVsInputMatrices;COLLECTION;0;matscan_predictions</outputs>
<parameters>
<dataType>urn:lsid:biomoby.org:objectclass:MEME_Text</dataType>
<description />
<id>urn:lsid:biomoby.org:serviceinstance:genome.imim.es,runMemeText;runMemeText;SIMPLE;0;meme_predictions
</id>
<name>meme_predictions</name>
<operation>urn:lsid:biomoby.org:serviceinstance:genome.imim.es,runMemeText;runMemeText</operation>
<paramIndex>1</paramIndex>
<type>Simple</type>
</parameters>
<parameters>
<dataType>urn:lsid:biomoby.org:objectclass:Matrix</dataType>
<description />
<id>urn:lsid:biomoby.org:serviceinstance:genome.imim.es,runMatScanGFFCollectionVsInputMatrices;runMatScanGFFCollectionVsInputMatrices;COLLECTION;1;motif_weight_matrices</id>
<name>motif_weight_matrices</name>
<operation>urn:lsid:biomoby.org:serviceinstance:genome.imim.es,runMatScanGFFCollectionVsInputMatrices;runMatScanGFFCollectionVsInputMatrices</operation>
<paramIndex>1</paramIndex>
<type>Array</type>
</parameters>
...
</workflowReturn>

And the response will contain:

- a list with the data types involved.
- a list with the operations involved.
- a list with the parameters involved (graph vertices).
- a list of edges.
- a list of inputs (parameter ids).
- a list of outputs (parameter ids).
- a boolean 'full' that indicates if the algorithm found a path from the source to the target.

The resulting graph contains all the possible 'shortest paths' to obtain an object of the target data type from an object of the source data type.

When full is false on the response, the graph will not be complete and it will let you all the possible paths to obtain an object of the target data type.

10 DISCOVERING PUBLISHED WORKFLOWS

At this section we will show the power of the Magallanes' workflow composition tool trying to discover a published workflow using Magallanes.

This workflow "aims to cluster a set of co-regulated genes in subsets of genes showing similar configurations of transcription factor binding sites" [1].

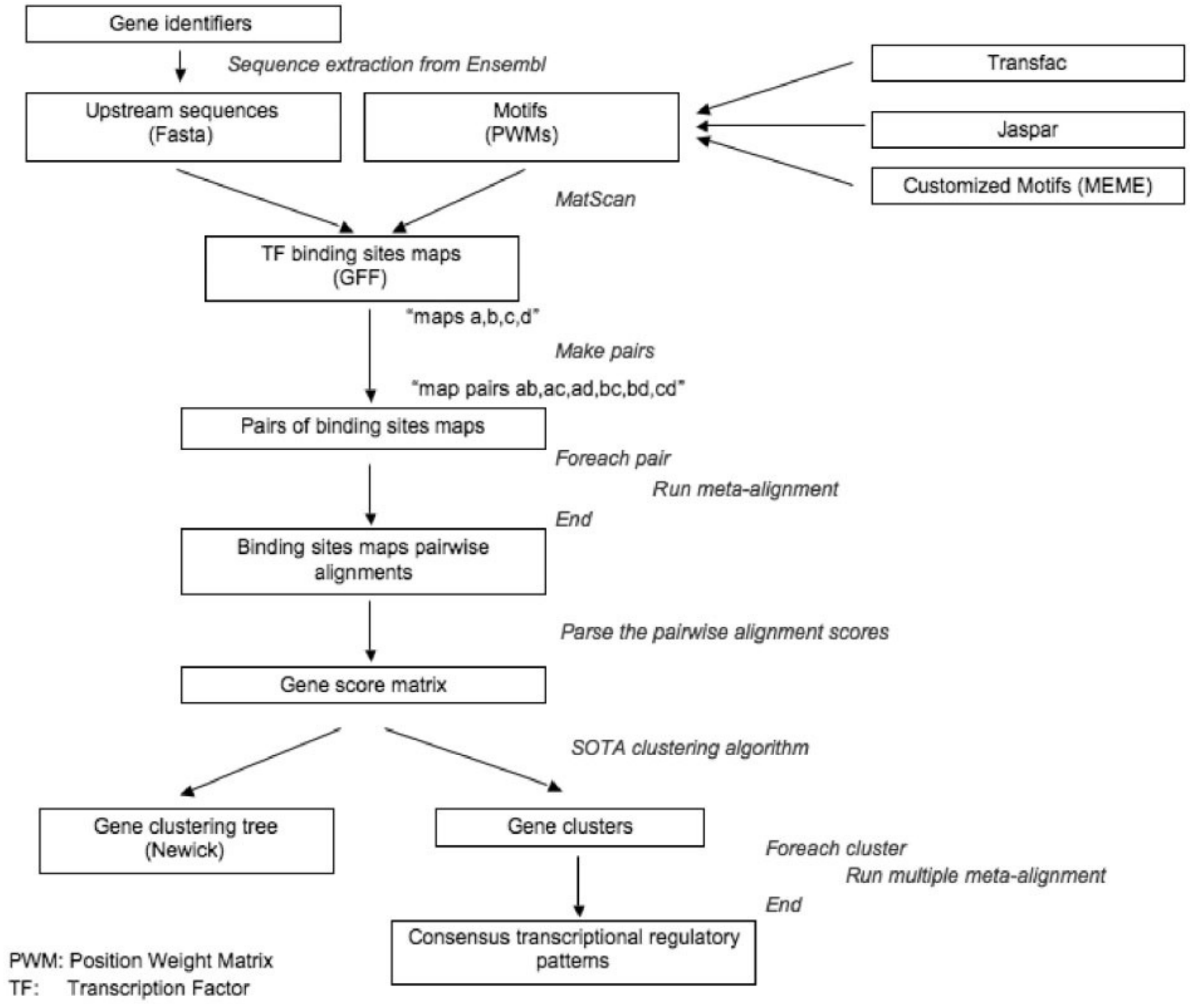

At first step we need to identify the source and target data types. The workflow gets multiple nucleic fasta sequences and returns a Newick clustering tree.

We will try to find the needed data types using Magallanes:

The first one will represent a collection of FASTA nucleic sequences.

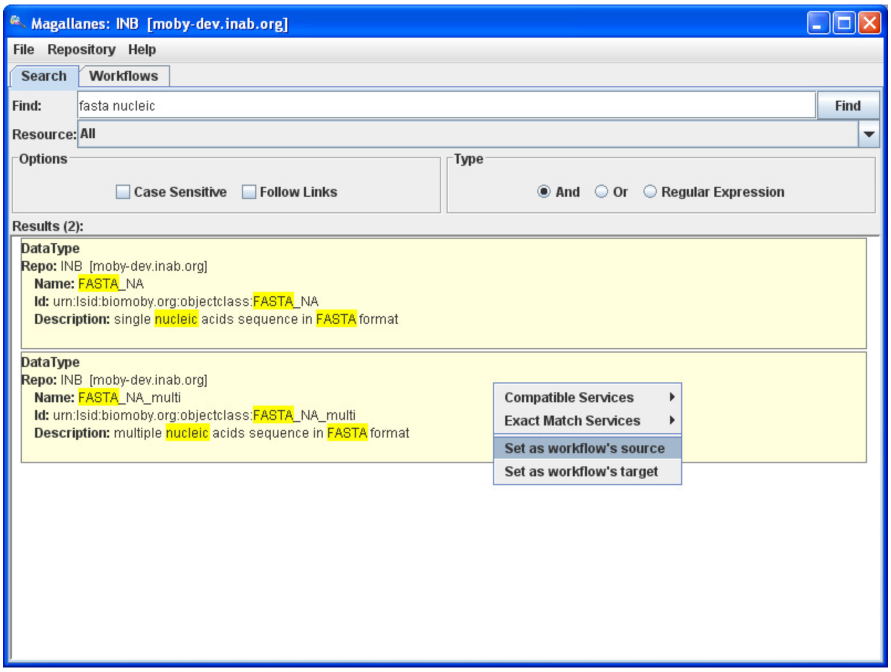

The Newick clustering tree can be represented using the Newick_Text data type:

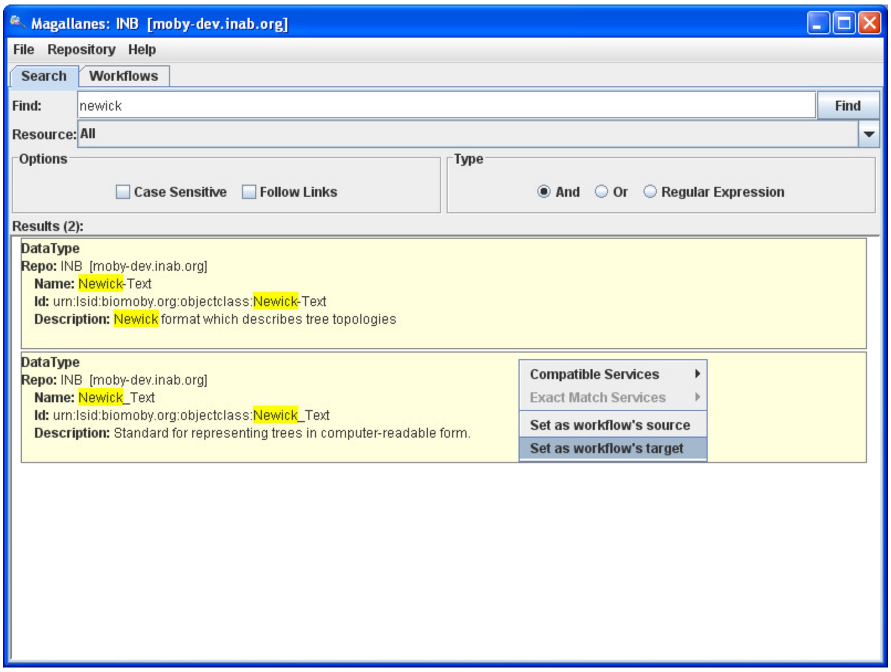

Now, you have the source and target data types on the Workflows tab and you are able to launch the generation task. For this example we will disable the Inheritance capability in order to reduce the resulting alternatives.

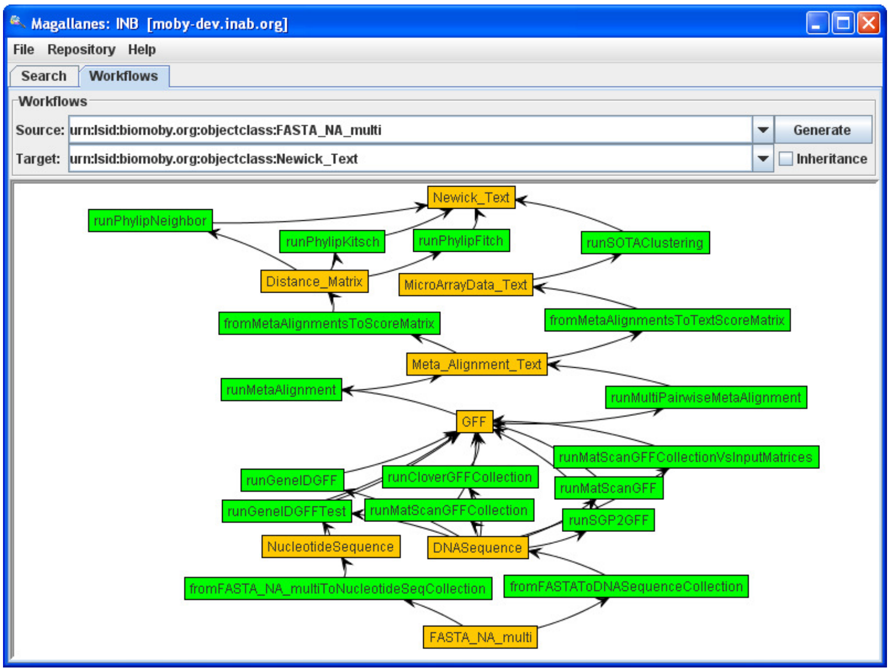

We have multiple alternative paths to go from FASTA_NA_multi to Newick_Text. We will select on each branch the desired service or datatype to obtain the searched workflow.
As we want to run a MatScan, we need to use the DNASequence branch on the first branch. We must select the 'fromFASTAToDNASequenceCollection' service by double clicking over it.

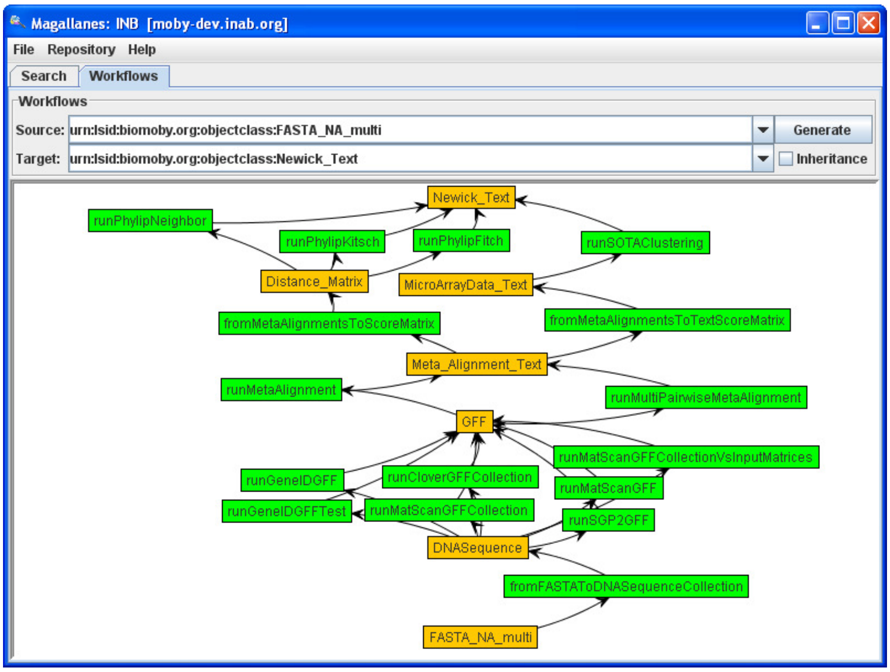

The next branch is the service used to obtain the GFF. As the original workflow uses MatScan, we will select the 'run-MatScanGFF' service.

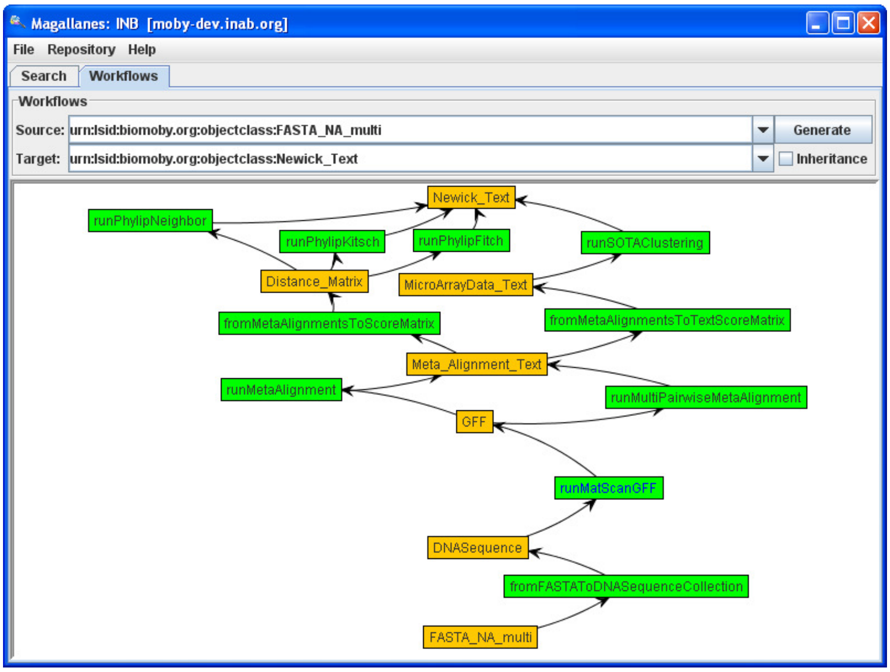

We will continue selecting the services used on the workflow: 'runMultiPairwiseMetaAlignment', 'fromMetaAlignmentsTo-TextScoreMatrix'.

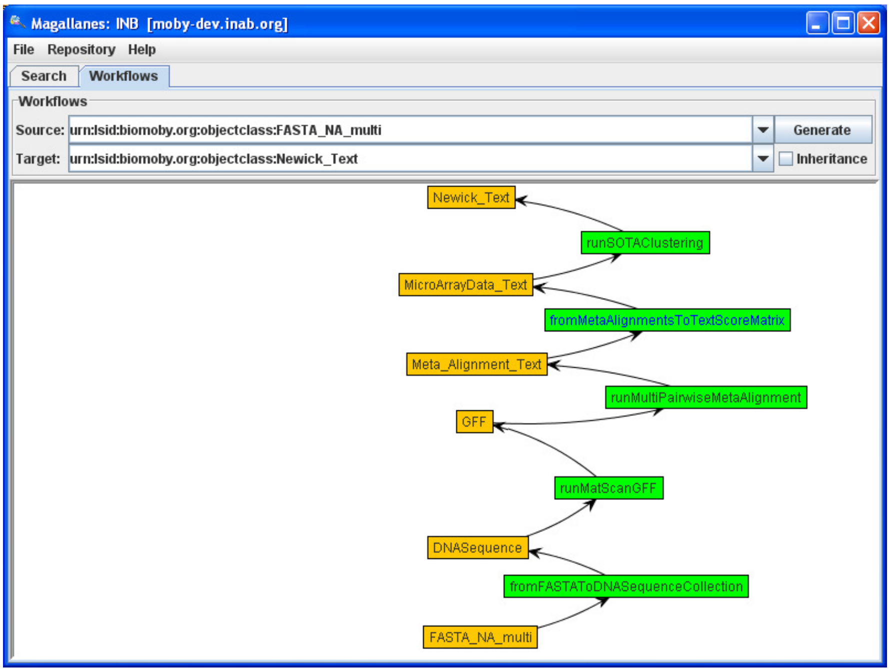

Now you can export the workflow and try to execute on Taverna. Export the workflow is as easy as right click and select export on the context menu.
The workflow imported on Taverna looks as follows:

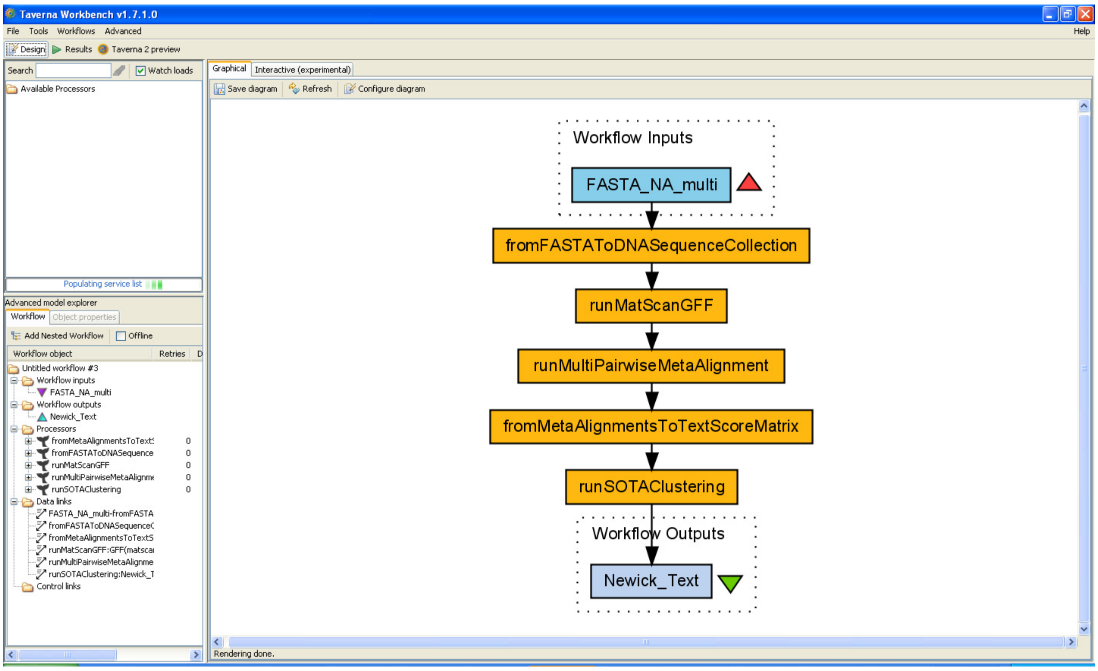

You can test the workflow using a set of FASTA sequences.

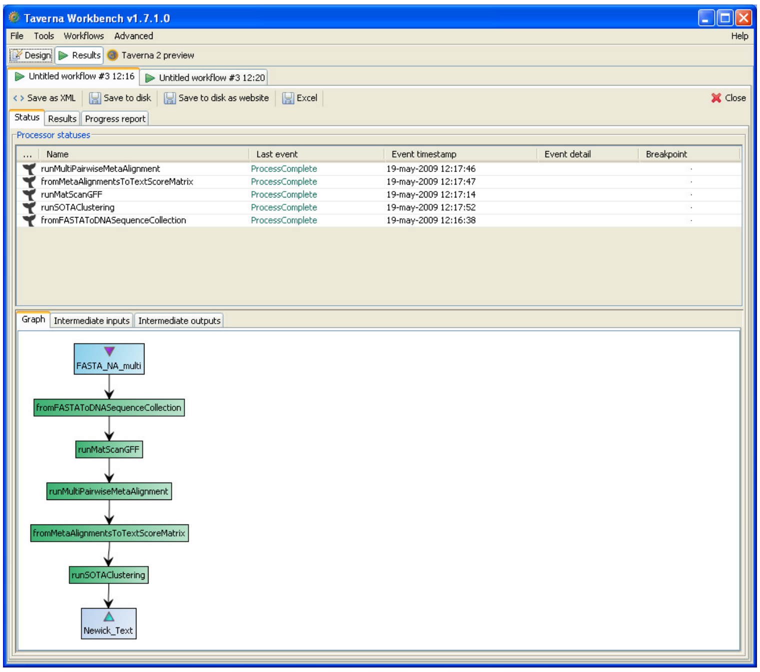

We can examine the resulted clustering using TreeView:

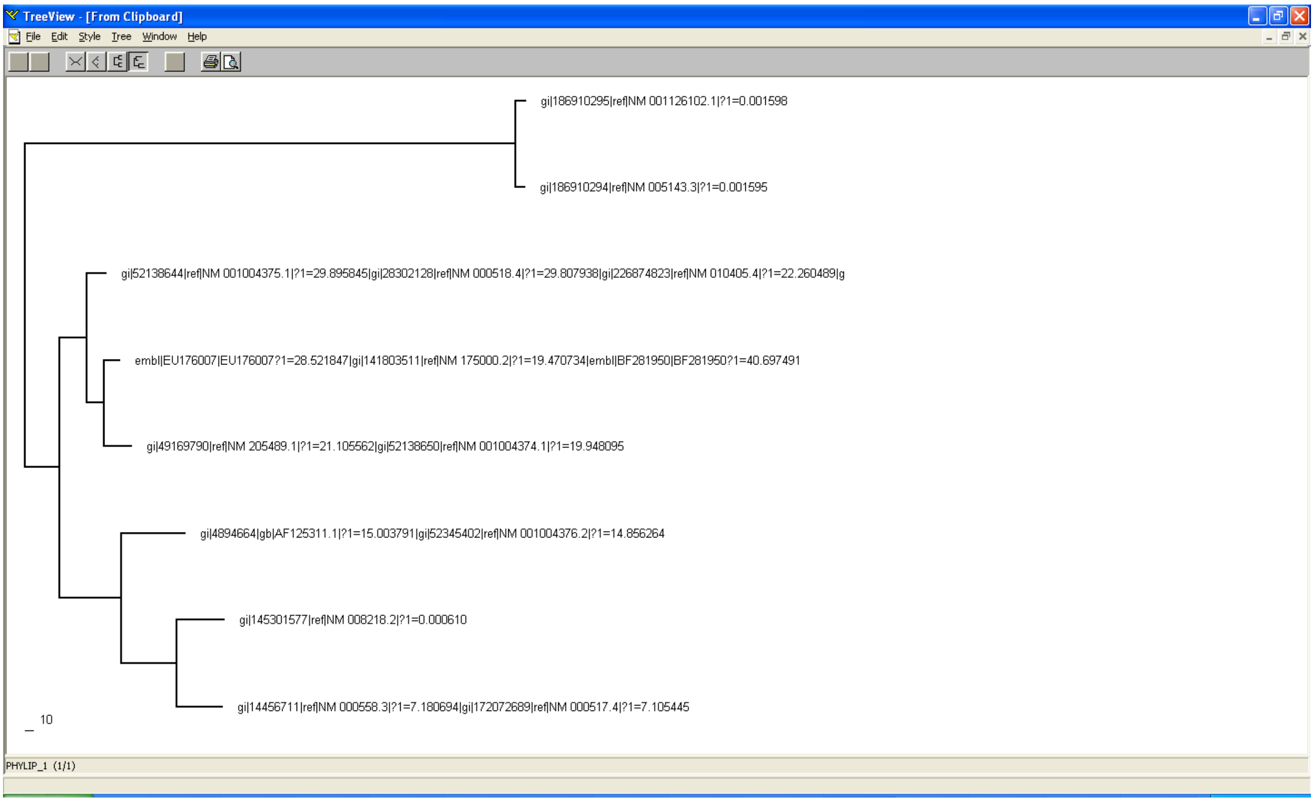

Analysis and Comments

The original WF published by Kerhornou and Guigó (2007) includes two additional beanshells (small pieces of software used to make simple data transformations):

1) Convert the output from 'runMultiMetaAlignmentGFF' into a image, The solution proposed by Magallanes do not includes this beanshell, but it ends with a Newick tree information which in fact represents the same information that can also be represented as an image.

2) Re-use the output from MatScan to avoid re-computing data to create the image.

We can conclude that the workflow proposed by Magallanes has the same functionality as it was published, Ruth the advantage of being obtained in few minutes instead of approximately 3 months as informed by the Authors (personal communication)

11 REFERENCES

[1] Kerhornou,A. and Guigó,R. (2007) BioMoby web services to support clustering of co-regulated genes based on similarity of promoter configurations. *Bioinformatics*, **23**(14), 1831-1833.
